# The transcriptomics profiling of blood CD4 and CD8 T-cells in narcolepsy type I

**DOI:** 10.3389/fimmu.2023.1249405

**Published:** 2023-11-23

**Authors:** Leila Khajavi, Xuan-Hung Nguyen, Clémence Queriault, Marianne Chabod, Lucie Barateau, Yves Dauvilliers, Matthias Zytnicki, Roland Liblau

**Affiliations:** ^1^ Toulouse Institute for Infectious and Inflammatory Diseases (Infinity), University of Toulouse, Centre National de la Recherche Scientifique (CNRS), L'Institut National de la Sante et de la Recherche Medicale (INSERM), Universite Paul-Sabatier de Toulouse (UPS), Toulouse, France; ^2^ Applied Mathematics and Informatics Unit of Toulouse (MIAT), Institut National de Recherche pour l'Agriculture, l'Alimentation et l'Environnement (INRAE), Toulouse, France; ^3^ Vinmec Institute of Applied Science and Regenerative Medicine, Vinmec Healthcare System and College of Health Sciences, VinUniveristy, Hanoi, Vietnam; ^4^ National Reference Center for Orphan Diseases, Narcolepsy, Idiopathic Hypersomnia and Kleine-Levin Syndrome, Department of Neurology, Gui-de-Chauliac Hospital, Centre Hospitalier Universitaire (CHU) de Montpellier, Montpellier, France; ^5^ Institute for Neurosciences of Montpellier (INM), University Montpellier, Montpellier, France; ^6^ Department of Immunology, Toulouse University Hospital, Toulouse, France

**Keywords:** narcolepsy type 1 (NT1), CD4 T-cell, CD8 T-cell, transcriptomics (RNA sequencing), autoimmunity, central nervous system

## Abstract

**Background:**

Narcolepsy Type I (NT1) is a rare, life-long sleep disorder arising as a consequence of the extensive destruction of orexin-producing hypothalamic neurons. The mechanisms involved in the destruction of orexin neurons are not yet elucidated but the association of narcolepsy with environmental triggers and genetic susceptibility (strong association with the HLA, TCRs and other immunologically-relevant loci) implicates an immuno-pathological process. Several studies in animal models and on human samples have suggested that T-cells are the main pathogenic culprits.

**Methods:**

RNA sequencing was performed on four CD4 and CD8 T-cell subsets (naive, effector, effector memory and central memory) sorted by flow cytometry from peripheral blood mononuclear cells (PBMCs) of NT1 patients and HLA-matched healthy donors as well as (age- and sex-) matched individuals suffering from other sleep disorders (OSD). The RNAseq analysis was conducted by comparing the transcriptome of NT1 patients to that of healthy donors and other sleep disorder patients (collectively referred to as the non-narcolepsy controls) in order to identify NT1-specific genes and pathways.

**Results:**

We determined NT1-specific differentially expressed genes, several of which are involved in tubulin arrangement found in CD4 (*TBCB, CCT5, EML4, TPGS1, TPGS2)* and CD8 (*TTLL7*) T cell subsets, which play a role in the immune synapse formation and TCR signaling. Furthermore, we identified genes (*GZMB, LTB* in CD4 T-cells and *NLRP3, TRADD, IL6, CXCR1, FOXO3, FOXP3* in CD8 T-cells) and pathways involved in various aspects of inflammation and inflammatory response. More specifically, the inflammatory profile was identified in the “naive” subset of CD4 and CD8 T-cell.

**Conclusion:**

We identified NT1-specific differentially expressed genes, providing a cell-type and subset specific catalog describing their functions in T-cells as well as their potential involvement in NT1. Several genes and pathways identified are involved in the formation of the immune synapse and TCR activation as well as inflammation and the inflammatory response. An inflammatory transcriptomic profile was detected in both “naive” CD4 and CD8 T-cell subsets suggesting their possible involvement in the development or progression of the narcoleptic process.

## Introduction

Narcolepsy Type I (hereafter referred to as NT1) is a rare sleep disorder arising as a consequence of the extensive destruction of orexin (also known as hypocretin)-producing hypothalamic neurons. It is characterized by excessive daytime sleepiness (EDS), fragmented nocturnal sleep, sleep paralysis, sleep-related hallucinations and cataplexy [Bassetti et al. (1)]. The prevalence of NT1 in Europe and North America is roughly one in two thousand individuals. NT1 is a chronic, life-long disorder which affects males and females at similar frequencies. Onset occurs in early childhood or adolescence with a higher incidence of the development of emotional (depression), cognitive (attention problems) and metabolic (obesity) disturbances over time [Barateau et al. (2); Mahoney et al. (3)].

Autopsies of NT1 patients revealed that over 90% of the orexin neurons in the lateral hypothalamus were undetectable [Peyron et al. (4)]. The mechanisms involved in the extensive destruction or silencing of orexin neurons are not yet elucidated [Seifinejad et al. (5)] but an immuno-pathological process has been implicated. Narcolepsy is polygenic in nature and exhibits a particularly strong association with the human leukocyte antigen (HLA) locus with ninety eight percent of NT1 patients carrying the HLA-DQB1*06:02 allele, resulting in an odds ratio of greater than 200 across ethnic groups. Weaker but significant associations were also found in HLA class I alleles and other immune-relevant loci, such as TCR*α* and *β* [Ollila et al. (6); Tafti et al. (7); Ollila et al. (8)].

Shortly after the H1N1 *influenza* pandemic in the winter of 2009-2010, there was a dramatic increase in new NT1 cases among children and adolescents (5-14 fold) as well as adults (2-7 fold), again with a strong association with the HLA-DQB1*06:02 allele [Sarkanen et al. (9)]. In Europe, this was linked to a particular H1N1 vaccine called Pandemrix (GlaxoSmithKline). Whereas in China, the observed increase (3 fold) in narcolepsy cases was among unvaccinated, yet supposedly infected, individuals [Han et al. (10)].

The current hypothesis is that microbial antigens (from an infection or vaccination), which share similarities with self-antigens from orexin neurons, are taken up by dendritic cells in peripheral tissues. These dendritic cells mature and migrate to local lymph nodes where they present epitopes to T-cells. These activated, cross-reactive T-cells then migrate across the blood-brain barrier (BBB) to the central nervous system (CNS) [Liblau et al. (11)]. In the brain, an (auto)immune response mediated by these activated T-cells contribute to the selective destruction, dysregulation or silencing of orexin-producing neurons in the hypothalamus [Seifinejad et al. (5)]. It has been shown that nearby, non-orexinergic neurons are left largely unharmed [Julkunen and Partinen (12)], with the exception of corticotropin-releasing hormone (CRH) neurons in the paraventricular nucleus [Shan et al. (13)].

To test this hypothesis, the peripheral blood mononuclear cells (PBMCs) of NT1 patients and age-, gender- and HLA-matched healthy controls have been analyzed via high-dimensional mass cytometry [Hartmann et al. (14)]. This study revealed that NT1 patients display multifaceted immune activation in CD4 and CD8 T-cells dominated by elevated levels of B cell supporting cytokines. Additionally, T-cells from narcolepsy patients showed markedly increased production of the proinflammatory cytokines IL-2 and TNF-*α*, indicative of inflammatory processes in the pathogenesis of this enigmatic disease. Latorre et al. identified an increased frequency of autoreactive CD4 T-cells in the blood of narcoleptic patients using a sensitive T-cell library method [Latorre et al. (15)]. They polyclonally expanded memory T-cell populations and detected CD4 T-cells reacting to epitopes along the entire prepro-orexin sequence. In a few NT1 patients, CD8 T-cells reacting to prepro-orexin were also detected, suggesting that NT1 may arise from an interaction of CD4 and CD8 T-cells. Another study identified an autoimmune process targeting a post-transcriptionally modified orexin peptide without detectable cross-reactivity with the flu proteins (HA and NP) of the pandemic H1N1 strain [Luo et al. (16)].

Here, we set out to identify NT1-specific differentially expressed genes and pathways by comparing the transcriptome of CD4 and CD8 T-cell subsets (naive, effector, effector memory, central memory) isolated from PBMCs of NT1 patients as well as healthy donors and individuals suffering from other sleep disorders. This endeavor should help determine the specific contributions of each T-cell compartment and subset to the development and progression of narcolepsy by identifying relevant genes and pathways.

## Materials and methods

### Patient/Donor summary

NT1 patients as well as patients with complaint of daytime sleepiness but with normal orexin levels were characterized at the National Reference Center for Orphan Sleep Disorders (Guide-Chauliac Hospital, Montpellier, France). NT1 was diagnosed following the guidelines of the International Classification of Sleep Disorders [American Academy of Sleep Medicine (17)] as follows: presence of excessive daytime sleepiness (EDS), presence of cataplexy, mean sleep latency during the multiple sleep latency test of less than 8 minutes, two or more sleep onset REM periods (SOREMPS) as well as cerebrospinal fluid (CSF) orexin levels of less than 110 pg/mL. Persons with other sleep disorder (OSD) had CSF orexin levels greater than 220 pg/mL (**Table 1**). All patients had blood drawn between 7 and 8am with all having fasted overnight in the sleep laboratory after a polysomnography recording. Blood samples from both NT1 and OSD patients were shipped within 24 hours of blood draw to the Toulouse Institute for Infectious and Inflammatory Diseases (Toulouse, France) where PBMCs were extracted and cryopreserved in liquid nitrogen. Healthy donor samples were drawn in Toulouse where PBMCs were extracted and cryopreserved. All NT1 patients and healthy controls were selected to carry the HLA-DQB1*06:02 susceptibility allele.

**Table 1:** Summary of patients and controls involved in the study.

Healthy Donors (HD)	
N	11
Age (years)	31 (18-46)
Sex (male/female)	5/6
HLA-DQB1*06:02 positive	11
Narcolepsy Type 1 (NAR)	
N	11
Age (years)	31 (15-49)
Sex (male/female)	5/6
HLA-DQB1^*^06:02 positive	11
Prior Pandemrix Vaccination	7
Disease Duration (months)	72 (35-153)
CSF orexin levels < 110 pg/mL	11
Other Sleep Disorder (OSD) ^*^	
N	11
Non-Specific Hypersomnolence	5
Idiopathic Hypersomnia	5
Narcolepsy Type 2 (NT2)	1
Age (years)	30 (13-45)
Sex (male/female)	5/6
CSF orexin levels (260-360 pg/mL)	7/11 tested

*Patients with complaint of excessive daytime sleepiness.

Eleven NT1 patients (five males and six females) between the ages of 15 to 49 and a disease duration of 35 to 153 months (at time of sampling) were selected. Eleven age-, sex- and HLA-matched healthy donors as well as eleven age- and sex-matched individuals with OSD were selected (**Table 1**). The eleven OSD patients consisted of five individuals diagnosed with non-specific hypersomnolence, five individuals diagnosed with idiopathic hypersomnia and one patient diagnosed with narcolepsy type 2. All individuals involved gave written informed consent to take part in the research program which was approved by the ethics committee, Comite´ de Protection des Personnes (2018-A00703-52, SOMNOBANK).

### T-cell subset sorting scheme

Peripheral blood from persons with NT1 or other sleep disorders was drawn at the National Reference Center for Orphan Sleep Disorders (Gui-de-Chauliac Hospital, Montpellier, France). Cryopreservation of PBMCs from patients and healthy volunteers was performed at INSERM U1291 (Toulouse, France) within 24 hours of blood collection. On average, 20-30 mL of blood was drawn from each patient/matching control into heparinized tubes. The blood was diluted three-fold with PBS. The diluted blood was then carefully layered onto the Ficoll density gradient medium and centrifuged for 20 minutes at 2000 rpm without brake. The PBMC layer was then collected, and the cells were washed with PBS and counted. Cells were resuspended at a concentration of 10x10^6^ PBMC/mL in a freezing medium of fetal bovine serum supplemented with 10% DMSO and then stored at -80C for 24 hours before transferring to liquid nitrogen. Cryopreserved PBMCs were stored in liquid nitrogen for 1 to 55 months (mean: 24.2 months).

For thawing, PBMC vials were held on the surface of a 37C water bath for about one minute and then resuspended gently in 10 ml of pre-warmed RPMI medium supplemented with 1% L-glutamine (100x), 1% sodium pyruvate (100mM), 1% non-essential amino acids (100x), 1% penicillin-streptomycin (10,000 U/ml), 1% HEPES (1M) and 5% filtered and inactivated human AB-serum. Viability of the T cells was assessed after thawing by flow cytometry using the Fixable Viability dye (ThermoScientific) after gating on CD3+ single cells. The viability ranged from 70.3 to 98.8% (mean: 93.6%). If the cell count thresholds were not met for a given patient, another tube of cryopreserved PBMCs from that patient was thawed and combined with the first tube prior to cell sorting.

Cell sorting was performed using a BD FACSAria Fusion with an antibody panel against CD3 (BD Horizon), CD4 (BD BioSciences), CD8*α* (BD Pharmingen), CCR7 (BD Horizon) and CD45RA (BD BioSciences) as well as eBioscience Fixable Viability Dye AF488. The Tcell subset sorting scheme (**Supplementary Figures 1A, C**) used CCR7 and CD45RA cell surface markers to identify the four T-cell subset for each individual sample. Post sorting, the cell number and viability were analyzed with the FlowJo software (**Supplementary Figures 1B, D**).

### RNA sequencing

Total RNA was extracted from sorted cells from each sample separately using the RNeasy Micro Kit (Qiagen). RNA quality and quantity were evaluated prior to RNA sequencing of the individual samples using the Eukaryote Total RNA Pico assay (Agilent). All samples were high quality with RINs ≥ 7 yet low quantity, ranging in concentration from 0.5 to 5 ng/µL for the CD4 compartment and 0.1 to 3 ng/µL for the CD8 compartment. Library preparation (SMART-seq v4, Takara Bio) and RNA-sequencing (Illumina NovaSeq) were performed by the Genomics Facility Basel in Switzerland. Both datasets were sequenced at a minimum of 50 million reads per sample. The CD8 compartment generated 50-bp paired-end reads resulting in an average of 50% unambiguously assigned fragments (range of 23 to 65%). The CD4 compartment generated 100-bp paired-end reads resulting in an average of 60% unambiguously assigned fragments (range of 26 to 78%).

### Bioinformatics analysis

All samples were analyzed separately through the quality control, mapping and quantification steps. Raw sequences were quality checked using FastQC [Andrews (18)] (version v0.11.2) and FastqScreen [Wingett and Andrews (19)] (version v0.11.4) prior to aligning to the Homo sapiens primary genome sequence (Gencode: GRCh38, v27) using STAR [Dobin et al. (20)] (version v2.6.0c). FastQC and PicardTools [Broad Institute (21)] (version v2.17.3) were used to assess the mapping quality. RSEM [Li and Dewey (22)] (v1.3.0) was used to generate the expression matrix. The principal component analysis (PCA) was performed on each CD4 and CD8 T-cell compartments to visualize the global distribution of CD4 and CD8 T-cell subsets and identify potential outliers. Both FeatureCounts [Liao et al. (23)] (v1.4.5) and mmQuant [Zytnicki (24)] were used to generate count estimations for each sample to produce metrics for quality control. Samples with low counts (below 20% successfully aligned fragments) that displayed a non-uniform distribution (clustering far away from other biological replicates) were removed prior to differential expression analysis.

All read counts where then pulled together into one expression matrix. This expression matrix (post outlier removal) was then analyzed with edgeR robust options [Robinson et al. (25); Zhou et al. (26)] (v3.26.8) in the R environment [Morandat et al. (27); R Core Team (28)] (version v3.6.3, BioConductor version v3.9 [Gentleman et al. (29); Huber et al. (30)]) to identify the differentially expressed genes (DEGs).

Counts were variance stabilized (DESeq2 v1.24.0, [Love et al. (31)]) prior to generating the PCA plots (ggplot2 v3.3.5, R package) and log transformed prior to generating the heatmaps (pHeatmap v1.0.12, R package). The UpSet plots were made using the ComplexHeatmap [Gu et al. (32)] (v2.0.0) and ComplexUpset (v1.3.0) R packages. Volcano plots were generated using the EnhancedVolcano [Blighe et al. (33)] R package (v1.2.0). Boxplots were generated using ggplot2 with Wilcoxon statistic using ggpubr R package (v0.4.0). The mapping coverage of each gene was then interrogated via Integrative Genome Viewer [Robinson et al. (34)] (IGV, v2.8.0). The protein-protein interaction plot was generated using STRING database (STRINGdb, v11.5) [Szklarczyk et al. (35)].

Gene set variation analysis (GSVA) was performed using the normalized count matrix for the CD4 and CD8 T-cell subsets in the R environment (GSVA v1.44.5, [Hänzelmann et al. (36)]). Briefly, GSVA is an unsupervised method which performs per sample gene set enrichment analysis. The molecular signature database (MSigDB) was used, focusing on the immunologic signature gene sets (C7). A pairwise Wilcoxon t-test was applied to the acquired GSVA results, comparing NT1 to healthy donors as well as NT1 to other sleep disorders. Only GSVA results with a p-value ≤ 0.05 for both pairwise analyses were retained for further interrogation.

### Analysis strategy

In order to focus on NT1-specific DEGs and exclude genes that may be differentially expressed due to the dysregulation of the sleep/wake cycle, the narcolepsy (NAR) data-set was compared against two controls: healthy donors (HD) and other sleep disorders (OSD), collectively referred to as non-narcolepsy (nonNAR). Bluntly grouping the nonNAR data-sets would overestimate the variance of this grouped set since we observed a clear difference between the HD and OSD data-sets. Another reason for which this method was favored was to avoid any potentially confounding effects the different blood handling and shipment conditions may have on the downstream transcriptomics analysis.

To this end, we designed a model where each group (NAR, OSD and HD) parameters are estimated and leveraged edgeR’s contrast method in order to compare NAR vs nonNAR, using weights for the generalized linear model of +1 for NAR and -0.5 for each control group (OSD and HD) with a false discovery rate (FDR) cutoff of 0.05. Each T-cell subset was analyzed separately in order to focus on subset-specific contributions. Wilcoxon test was applied to each DEG, comparing NAR versus HD and NAR versus OSD. DEGs with a Wilcoxon p-value ≤ 0.05 for both pairwise comparisons were retained as NT1-specific DEGs.

### Data availability

The transcriptomic data is available on NCBI GEO under accession number GSE240851.

## Results and discussion

### Global distribution of blood CD4 and CD8 T-cell subsets

**Figure 1:**
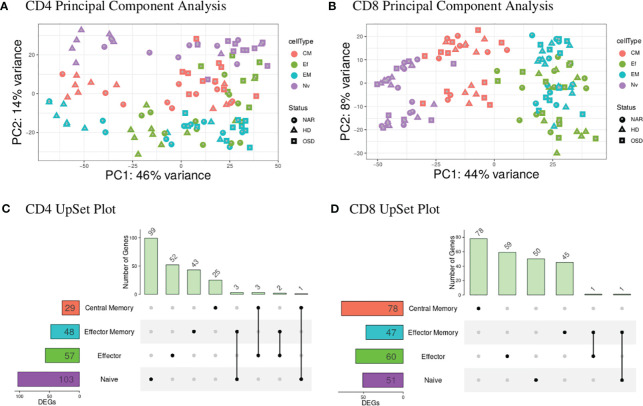
**(A)** CD4 PCA displays the highest variance (PC1) of 46% most likely attributed to the disease status of the individuals tested with healthy donors (triangles) predominantly to the left of the graph and narcolepsy (circles) and OSD (squares) samples clustered to the right of the graph. PC2 shows a 14% variance attributed to the T-cell subsets with naive (purple) at the top, central memory (red) in the middle and effector (green) and effector memory (blue) samples at the bottom. **(B)** CD8 PCA displays the highest variance (PC1) of 44% attributed to the T-cell subsets with naive (purple) at the left, central memory (red) in the middle and effector (green) and effector memory (blue) samples to the right. PC2 displays 8% variance attributed to the gender of the individuals. **(C)** CD4 UpSet plot summarizing the differentially expressed genes across the four CD4 T-cell subsets. The T-cell subsets are listed on the y-axis with the numbers in the box representing the number of DEGs. Individual black dots indicate unique genes to the given subset while black dots connected with a black line indicate shared genes between the implicated subsets. The histogram at the top displays the number of genes belonging to the group or groups indicated by the black dots and dots connected by a line. **(D)** CD8 UpSet plot summarizing the differentially expressed genes across the four CD8 T-cell subsets. (CM, Central Memory; EM, Effector Memory; Ef, Effector; Nv, Naive; DEGs, differentially expressed genes).

A global representation of CD4 and CD8 data-sets are shown in the PCA plots (**Figures 1A, B**). The UpSet plots (**Figures 1C, D**) summarize DEGs in the four T-cell subsets indicating the number of DEGs restricted to a given subset and genes that are shared by several subsets for CD4 and CD8 T-cell compartments.

The blood CD4 T-cell subsets (**Figure 1A**) show a global distribution dependent on disease status where the HD samples are separating away from both NAR and OSD samples. This is also reflected in the CD4 UpSet plot (**Figure 1C**) where there is a greater number of shared DEGs between the CD4 T-cell subsets, corresponding to the clustering of these subsets in the PCA plots. The larger the number of shared DEGs between subsets, the higher the concordance between the subsets.

The blood CD8 T-cell subsets (**Figure 1B**), in contrast, show a global distribution with most of the variance based on T-cell subsets. The T-cell subsets create a separation gradient of naive, central memory, effector and effector memory. This separation is as expected with the effector and effector memory subsets overlapping (due to their high similarities and effector function) and the central memory subset (which lacks effector function) separating to the middle of the gradient between the naive and effector/effector memory subsets [Seder and Ahmed (37)].

The differences in the CD4 and CD8 global distribution visible in the two PCA plot may be explained by the relative frequencies of the different T-cells and their subsets present in healthy controls and those suffering from viral infections. Sedar and Ahmed reported that the frequency of CD4 T-cell subsets (from PBMC populations of healthy individuals compared to those with a chronic viral infection) remained fairly stable when comparing the two disease statuses [Seder and Ahmed (37)]. This may explain why the largest variance in our CD4 data-set is disease-associated and not subset-associated. In contrast, the frequency of CD8 T-cell subsets change when comparing the two disease statuses, which may explain why the largest variance in our CD8 T-cell data-set is associated with the T-cell subsets.

### CD4 T-cell subsets

**Figure 2:**
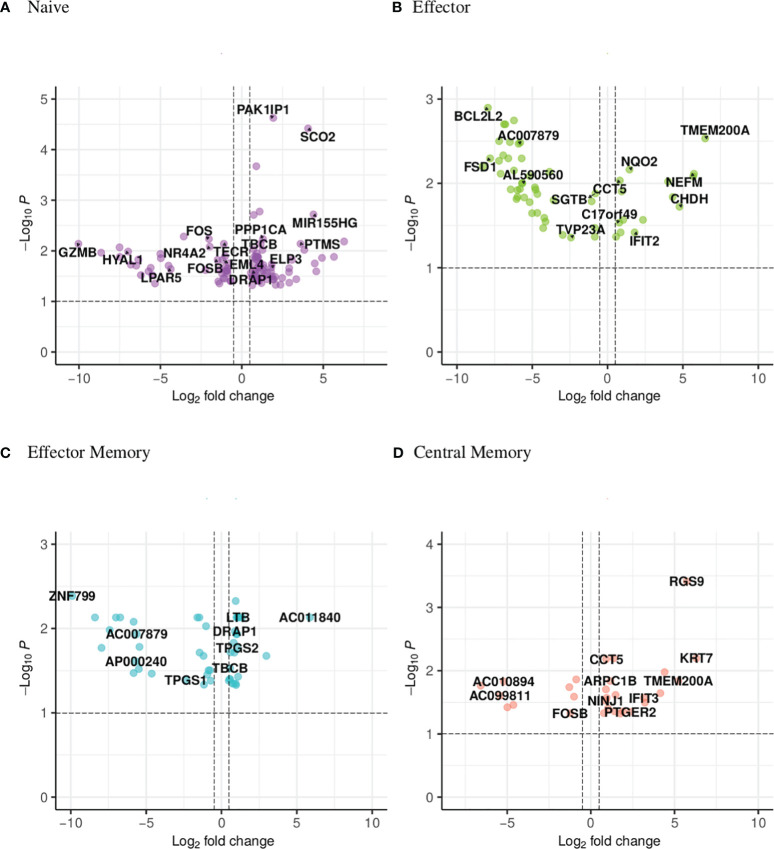
Volcano plot showing the distribution of the DEGs of narcoleptic patients versus nonnarcoleptic individuals in the **(A)** naive, **(B)** effector, **(C)** effector memory and **(D)** central memory CD4 T-cell subsets. Along the x-axis is the log2 fold-change and along the y-axis is the adjusted p-value.

The UpSet plot in **Figure 1C** summarizes the DEGs in the four CD4 T-cell subsets. Notably, the shared DEGs are congruently down- or up-regulated in the different CD4 T-cell subsets from NT1 patients as compared to the two control groups. There are three genes (DRAP1, TBCB, EML4) shared between the naive and effector memory subsets; three genes (TMEM200A, CCT5, SGTB) shared between the effector and central memory subsets; two genes (C17orf49 and AC007879) shared between effector and effector memory subsets and one gene (FOSB) shared between naive and central memory subsets. The heatmaps (**Supplementary Figures 2**-**5**) and volcano plots (**Figure 2**) were generated, per subset, using the genes summarized in the UpSet plot. The GSVA plots (**Supplementary Figure 6**) were generated, per subset, using the normalized gene counts.

### Genes shared between CD4 subsets

**Figure 3:**
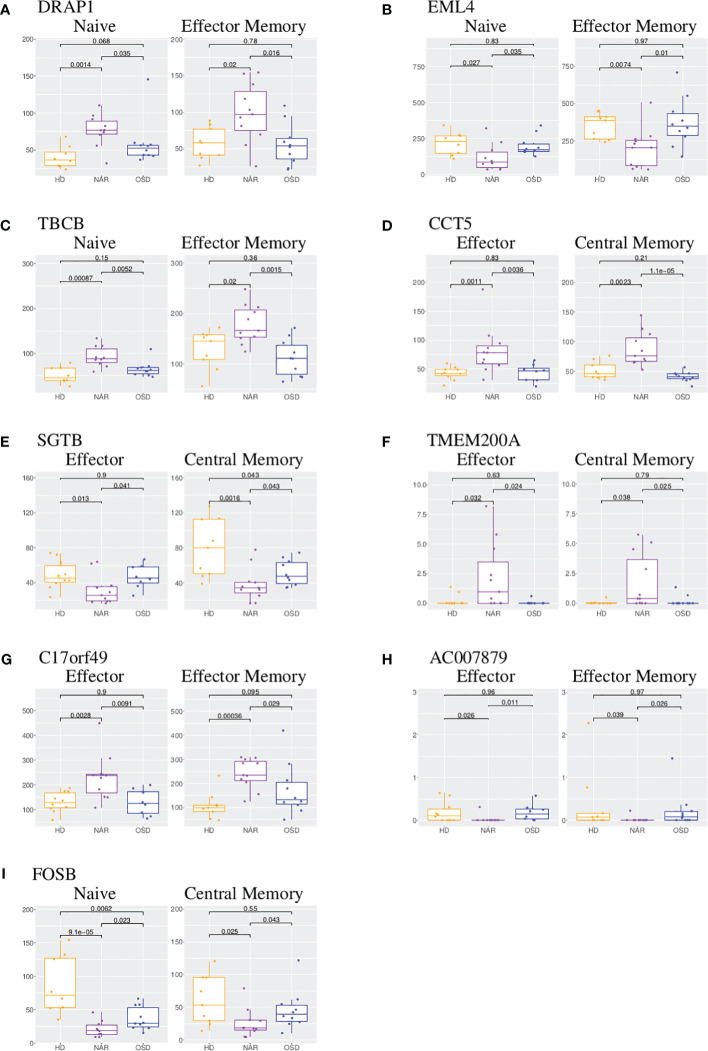
Boxplot of DEGs shared between CD4 T-cell subsets. **(A)** Boxplots of DRAP1 shared in CD4 naive and effector memory subsets. **(B)** Boxplots of EML4 shared in CD4 naive and effector memory subsets. **(C)** Boxplots of TBCB shared in CD4 naive and effector memory subsets. **(D)** Boxplots of CCT5 shared in CD4 effector and central memory subsets.**(E)** Boxplots of SGTB shared in CD4 effector and central memory subsets. **(F)** Boxplots of TMEM200A shared in CD4 effector and central memory subsets. **(G)** Boxplots of C17orf49 shared in CD4 effector and effector memory subsets. **(H)** Boxplots of AC007879 shared in CD4 effector and effector memory subsets. **(I)** Boxplots of FOSB shared in CD4 naive and central memory subsets. Each boxplot displays the counts-per-million (CPM) along the y-axis and disease status (HD, NAR, OSD) along the x-axis with the p values of the Wilcoxon test displayed at the top.

DRAP1 (DR1-associated co-repressor) functions as a transcriptional repressor and is upregulated in both NAR naive and effector memory subsets (**Figure 3A**). It has been linked to narcolepsy in a previous report studying the comparative gene expression profiling via microarray of narcolepsy patients compared to healthy donors [Bernardini et al. (38)]. In the study (analyzing total RNA extracted from whole blood), DRAP1 was reported as down-regulated in NAR samples in comparison to healthy donors. EML4 (Echinoderm microtubule associated protein-like 4) gene is involved in the T-cell receptor signaling pathway and is downregulated in both naive and effector memory subsets (**Figure 3B**).

TBCB (Tubulin folding cofactor B) is also upregulated in both naive and effector memory CD4 T-cells (**Figure 3C**). Tubulins and actins are reorganized in T-cells at the immune synapse; upregulation of TBCB may result in alterations in T-cell activation and signaling [Martín-Cófreces et al. (39, 40)]. CCT5 (Chaperonin containing TCP1 subunit 5) is upregulated in both effector and central memory subsets (**Figure 3D**) and is involved in neuropathy related pathways. It has been reported that CCT5 controls the polarization of tubulin at the immune synapse as well as the metabolic status of T-cells which affect T-cell activation [Martín-Cófreces et al. (40)]. A coordinated increase of CCT5 and TBCB has been reported upon T-cell activation [Martín-Cófreces et al. (40)] which may imply a coordinated potential function at the immune synapse upon T-cell activation.

SGTB (small glutamine rich tetratricopeptide repeat containing beta), down-regulated in both effector and central memory subsets (**Figure 3E**), is associated with neuronal apoptosis after neuroinflammation [Cao et al. (41)] and has been proposed as a biomarker of Giant Cell Arteritis in a longitudinal expression profiling of CD4 and CD8 T-cells [De Smit et al. (42)]. TMEM200A (transmembrane protein 200A) is significantly upregulated in NAR effector and central memory subsets (**Figure 3F**). The upregulation of TMEM200A has been reported in a study focusing on genes involved in the inhibition of the immune synapse [De Smit et al. (42)]. The two genes (C17orf49 and AC007879) shared between effector and effector memory subsets currently do not have a function associated with them. C17orf49 is up-regulated in both effector and effector memory subsets (**Figure 3G**) while AC007879 is down-regulated in these two subsets (**Figure 3H**).

FOS (down-regulated in NAR naive subset), FOSB (down-regulated in NAR naive and central memory subsets (**Figure 3I**)), PPP1CA (upregulated in NAR naive subset) and RGS9 (upregulated in NAR central memory subset) are all involved in the amphetamine addiction pathway. FOS (Fos proto-oncogene) and FOSB (Fosb proto-oncogene) are members of the Fos gene family which are implicated as regulators of cell proliferation, differentiation and transformation. Both FOS and FOSB were reported to be regulated by orexin-A stimulation [Koesema and Kodadek (43)]. PPP1CA (protein phophatase 1 catalytic subunit alpha) is involved in the regulation of cellular processes such as cell division and protein synthesis as well as long-term synaptic plasticity. RGS9 (Regulator of G-protein signaling 9) has been shown to play a role in chemokine-induced lymphocyte migration [Agenès et al. (44)].

### CD4 T-cell subset-specific DEGs

#### CD4 naive subset

**Figure 4:**
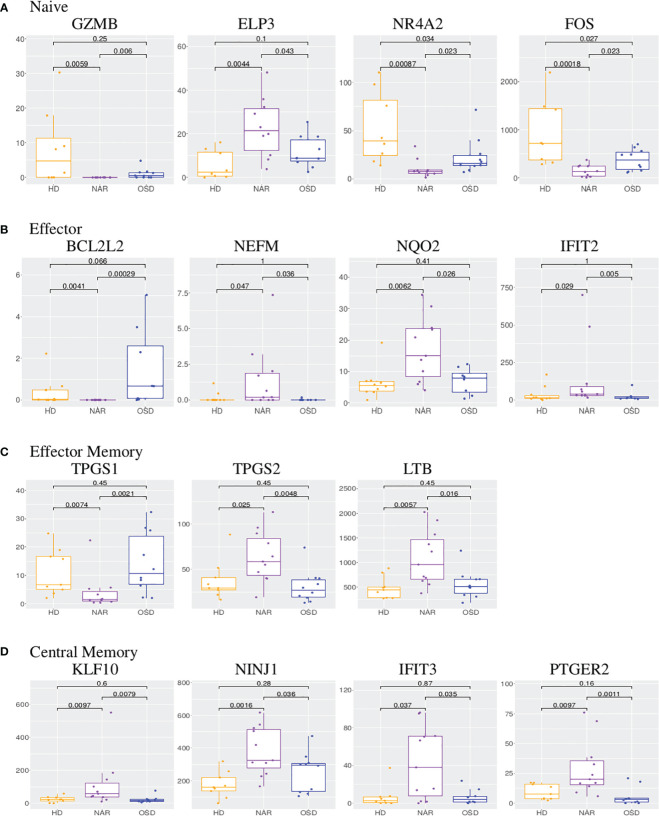
**(A)** Boxplots of GZMB, ELP3, NR4A2 and FOS in CD4 naive subset. **(B)** Boxplots of BCL2L2, NEFM, NQO2 and IFIT2 in CD4 effector subset. **(C)** Boxplots of TPGS1, TPGS2 and LTB in CD4 effector memory subset. **(D)** Boxplots of KLF10, NINJ1, IFIT3 and PTGER2 in CD4 central memory subset. Each boxplot displays the counts-per-million (CPM) along the y-axis and disease status (HD, NAR, OSD) along the x-axis with the p values of the Wilcoxon test displayed at the top.

Based on the UpSet plot (**Figure 1C**), it could be ascertained that the naive subset is the most dynamic with 103 DEGS, 99 of which are unique to this subset. In the naive subset (**Figure 2A**; **Supplementary Figure 2**), granzyme B (GZMB) is down-regulated (**Figure 4A**) in NAR subjects versus the nonNAR controls (log_2_ fold change of -10). GZMB is a key effector molecule secreted by cytotoxic lymphocytes (NK, CD8, CD4) and involved in target cell apoptosis. The secretion of GZMB by regulatory CD4 T-cells (Tregs) is an inhibitory mechanism utilized to regulate the immune system in order to prevent autoimmunity and limit chronic inflammatory diseases [Juno et al. (45)]. Our sorting strategy does not allow for the discrimination of naive conventional CD4 T-cells from naive Tregs.

ELP3 (elongation acetyltransferase complex subunit 3), upregulated in NAR naive subset (**Figure 4A**), regulates the maturation of projection neurons. It has been reported that ELP3 expression increases in the course of T-cell activation, whereas ELP3-deficient T-cells exhibited reduced expansion [Lemaitre et al. (46)]. NR4A2 (nuclear receptor subfamily 4 group A member 2), down-regulated in NAR naive subset (**Figure 4A**), is a transcription factor regulating the differentiation and functions of T-cells [Odagiu et al. (47)]. It has been shown that NR4A2 promotes cytokine production by CD4 T-cells and plays a role in Treg development. Down-regulation or deletion of NR4A2 in T-cells inhibits or blocks the induction of Tregs, exacerbating an immune response [Odagiu et al. (47); Sekiya et al. (48)]. Higher expression levels of NR4A family members has been linked to a T-cell exhaustion phenotype [Flemming (49)].

The GSVA plot for the CD4 naive subset (**Supplementary Figure 6A**) shows upregulation in several pathways including Th1 versus Th17, IL-4 treated activated CD4 T-cells versus untreated CD4 T-cells, mir17 over-expression in activated CD4 T-cells versus mir17 knock-out and STAT3 knock-out versus wild-type CD4 T-cell treated with IL6 which indicate an activated and inflammatory profile rather than a bona fide “naive” profile. Both the Th1 versus Th17 and STAT3 knock-out versus wild-type CD4 T-cell pathways imply a deficiency in STAT3 function, whereas, at the mRNA level, the STAT3 gene is not differentially expressed in the analysis. This may imply that post-transcriptional or post-translational modifications could be at play, modifying the STAT3 function at the protein level.

#### CD4 effector subset

The heatmap for CD4 effector subset (**Supplementary Figure 3**) displays a striking number of genes that seem completely “turned off” in the NAR samples in comparison to the two control groups. The bulk of these down-regulated genes belong to the long non-coding RNA (lncRNA) class. This is intriguing since there is a much greater proportion of mRNA transcripts/genes in the human annotated genome than there are lncRNAs [Frankish et al. (50)]. In fact, using the Ensembl annotation file (GRCh38.gtf), there are roughly three times as many protein coding genes (22041) as there are lncRNA genes (7439). Our observation may imply a regulatory role of lncRNAs in the activation, maintenance, or function of CD4 effector T-cells, which is notably perturbed in NAR patients.

BCL2L2, down-regulated in NAR effector subset (**Figure 4B**), contributes to reduced cell apoptosis under cytotoxic conditions. NEFM (neurofilament medium chain), upregulated in NAR effector (**Figure 4B**) subset, is used as a biomarker of neuronal damage. NQO2 (Quinone Reductase 2), upregulated in NAR effector (**Figure 4B**) subset, is a cytosolic flavoprotein. Mutations in this gene have been associated with neurodegerative diseases. It has also been reported that NQO2 (as well as NQO1) regulate humoral and auto-immunity [Iskander et al. (51)]. IFIT2 (interferon induced protein with tetratricopeptide repeats 2), upregulated in NAR effector subset (**Figure 4B**), inhibits expression of viral messenger RNAs and can promote apoptosis. Interestingly, IFTI2 transcripts are preferentially expressed in a cluster of terminally differentiated cytotoxic effecter CD4 T-cells [Patil et al. (52)].

The GSVA plot for the CD4 effector subset (**Supplementary Figure 6B**) displays a more balanced distribution of up and down-regulated gene sets. Several gene sets are upregulated in conventional CD4 T-cells (Tconv) compared to regulatory CD4 T-cells (Tconv versus FOXP3 knock-out induced Treg, Tconv CD4 T-cells from lymph nodes versus Tregs, Tconv PPARG knock-out versus Treg), implying a deficient Treg response as expected in an autoimmune disease.

#### CD4 effector memory subset

Examining the genes in the heatmap (**Supplementary Figure 4**) and volcano plot (**Figure 2C**), it appears that the down-regulated genes predominantly belong to the lncRNA class. The zinc-finger genes viewed on the left panel of the volcano plot as well as the bottom of the heatmap are all involved in transcription regulation. TPGS1 (tubulin polyglutamylase complex subunit 1) is down-regulated while TPGS2 (tubulin polyglutamylase complex subunit 2) is up-regulated in NAR effector memory subset (**Figure 4C**). Both of these genes are involved in the post-transcriptional modification of tubulin (regulator of microtubule function) which affect the cytoskeletal rearrangements at the immune synapse [ Verhey and Gaertig (53)].

LTB (lymphotoxin beta), up-regulated in NAR effector memory (**Figure 4C**) subset, is an inducer of the inflammatory response system. LTB has been identified as a potential gene belonging to or identifying the CD4 tissue-resident memory (TRM) subset in the lung, post influenza infection or vaccination [Swarnalekha et al. (54)]. LTB has also been suggested to play a role in the development or function of CD4 cytotoxic T-cells [Patil et al. (52)].

In the GSVA plot for the CD4 effector memory subset (**Supplementary Figure 6C**), narcolepsy samples are down-regulated in the Howard PBMC gene set compared to non-narcolepsy samples. The Howard gene set is a set of genes down-regulated in PBMCs vaccinated with an inactivated H5N1 vaccine with AS03 adjuvant versus inactivated H5N1 vaccine with saline. Narcolepsy samples also show a very strong upregulation in two yellow fever (YF) vaccine gene sets ([Scherer et al. (55); Gaucher et al. (56)]) in which genes are reported as down-regulated in the PBMCs of adults exposed to the yellow fever vaccine.

#### CD4 central memory subset

KLF10 (kruppel like factor 10), upregulated in NAR central memory subset (**Figure 4D**), is a transcriptional repressor which plays a role in the regulation of the circadian clock. It is hypothesized to regulate the circadian expression of genes involved in lipogenesis, gluconeogenesis and glycosis in the liver. KLF10 has been reported as a transcription factor regulated by orexin-A stimulation [Koesema and Kodadek (43)]. KLF10 expression in CD4 T-cells plays a role in obesity and Treg function [Wara et al. (57)]. NINJ1 (nerve injury-induced protein-1), upregulated in NAR central memory subset (**Figure 4D**), is upregulated after nerve injury [Araki and Milbrandt (58)] and involved in inflammation, cell death [Kayagaki et al. (59)] and chemotaxis [Ifergan et al. (60)]. Upregulated expression of NINJ1 on the surface of T-cells has been linked to CNS inflammatory diseases such as multiple sclerosis [Liu et al. (61)].

IFIT3 (interferon induced protein with tetratricopeptide repeats 3), upregulated in NAR central memory (**Figure 4D**) subset, acts as an inhibitor of viral processes, cell migration and viral replication. It is believed to regulate the innate immune response via the IRF3 (interferon regulatory transcription factor 3) gene [Johnson et al. (62)]. PTGER2 (prostaglandin E receptor 2, also known as EP2), upregulated in NAR central memory (**Figure 4D**), is a receptor for prostaglandin E2. T-cell intrinsic EP2 signaling has been reported to be critical in the generation of pathogenic T*_H_* 17 cells (a subset of pro-inflammatory T helper cells) resulting in tissue inflammation [Lee et al. (63)].

In the GSVA plot for the CD4 central memory subset (**Supplementary Figure 6D**), narcolepsy samples are upregulated in a gene set comparing healthy controls to infants with an acute influenza infection (GSE34205) as well as two vaccination gene sets comparing vaccinated versus saline control participants [Matsumiya et al. (64); Erwin-Cohen et al. (65)]. Interestingly, the Erwin-Cohen gene set describes the gene set upregulated in the blood of vaccinated participants who received a live, attenuated Venezuelan equine encephalitis virus while in another gene set the genes are upregulated in neuron cell lines infected with western equine encephalitis virus (GSE16451). Narcolepsy samples are upregulated in both of these gene sets compared to nonnarcolepsy samples.

### CD8 T-cell subsets

**Figure 5:**
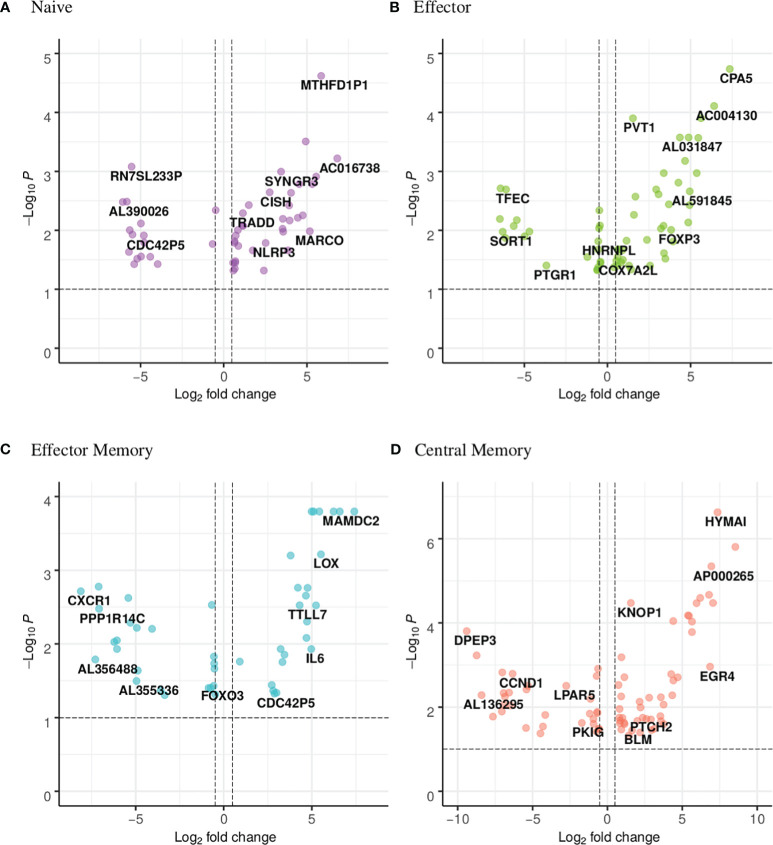
Volcano plot showing the distribution of the DEGs of narcoleptic patients versus nonnarcoleptic individuals in the **(A)** naive, **(B)** effector, **(C)** effector memory and **(D)** central memory CD8 T-cell subsets. Along the x-axis is the log2 fold-change and along the y-axis is the adjusted p-value.

The UpSet plot in **Figure 1D** summarizes the DEGs in the four CD8 T-cell subsets, indicating the number of genes restricted to a given subset or shared by several subsets. In the CD8 compartment, the central memory subset is the most dynamic with 78 unique DEGs. There is one gene (pseudogene CDC42P5) shared between naive and effector memory subsets as well as one gene (lncRNA HNRNPL) shared between effector and effector memory subsets. The heatmaps (**Supplementary Figures 7**-**10**) and volcano plots (**Figure 5**) were generated, per subset, using the genes summarized in the UpSet plot. The GSVA plots (**Supplementary Figure 11**) were generated, per subset, using the normalized gene counts. For the CD8 subsets, the general trend visible in the GSVA plots, in contrast to the CD4 subsets, is that the majority of the pathways appear to be down-regulated in narcolepsy samples in comparison with non-narcolepsy samples. There are also the presence of CD4 related pathways which may be explained by the fact that the two main classes of T-cells have largely overlapping gene expression.

### Genes shared between CD8 subsets

**Figure 6:**
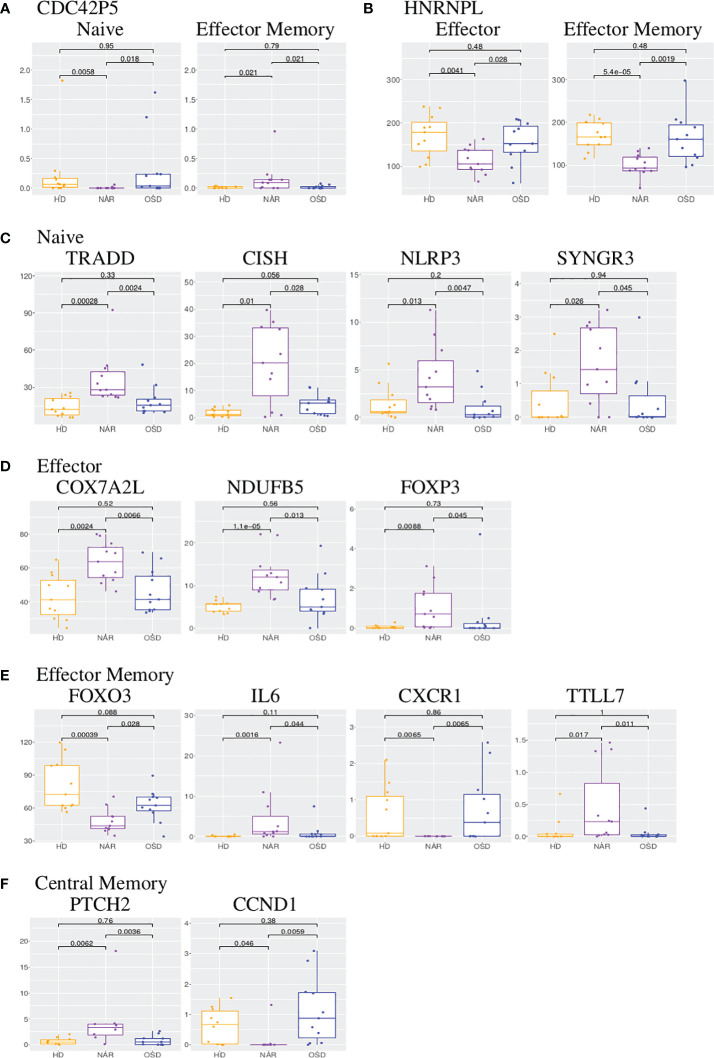
**(A)** Boxplots of CDC42P5 shared in CD8 naive and effector memory subsets. **(B)** Boxplots of HNRNPL shared in CD8 effector and effector memory subsets. **(C)** Boxplots of TRADD, CISH, NLRP3 and SYNGR3 in CD8 naive subset. **(D)** Boxplots of COX7A2L, NDUFB5 and FOXP3 in CD8 effector subset. **(E)** Boxplots of FOXO3, IL6, CXCR1 and TTLL7 in CD8 effector memory subset. **(F)** Boxplots of PTCH2 and CCND1 in CD8 central memory subset. Each boxplot displays the counts-per-million (CPM) along the y-axis and disease status (HD, NAR, OSD) along the x-axis with the p values of the Wilcoxon test displayed at the top.

CDC42P5 is a pseudogene shared between the CD8 naive and effector memory subsets (**Figure 6A**). Intriguingly, it is downregulated (fold change of -log_2_ 4.8) in the NAR naive subset yet upregulated in the NAR effector memory subset (fold change of log_2_ 2.9). There is no function currently associated with this pseudogene. HNRNPL (heterogeneous nuclear ribonucleoprotein L) is a lncRNA down-regulated in both effector and effector memory subsets (**Figure 6B**). This lncRNA plays a role in the formation, packaging, processing and function of mRNA and has been associated with Alzheimer’s disease [Dharshini et al. (66)].

### CD8 subsets-specific DEGs

#### CD8 naive subset

The following four genes are all upregulated in the NAR naive subset (**Figure 5A**; **Supplementary Figure 7**) and are immunologically relevant. TRADD (TNF-receptor 1-associated death domain, **Figure 6C**) reduces the recruitment of apoptosis-inhibitory proteins; its dysfunction can lead to auto-inflammation. CISH (cytokine inducible SH2 containing protein, **Figure 6C**) is a negative regulator of cytokine signaling of the SOCS family. CISH is induced by TCR stimulation and inhibits TCR signaling in CD8 T-cells [Palmer et al. (67)]. NLRP3 (NLR family pyrin domain containing 3, **Figure 6C**), a component of the inflammasome, plays a role in the regulation of inflammation, the immune response and apoptosis; it has been proposed as a prognostic marker for progressive multiple sclerosis [Malhotra et al. (68)]. SYNGR3 (synaptogyrin 3, **Figure 6C**) is a synaptic vesicle protein that interacts with the dopamine transporter. In a previous report studying synaptic stripping of neurons under T-cell attack, SYNGR3 was up-regulated in neurons in a murine model of encephalitis caused by CD8 T-cell targeting of antigenic neurons [Liberto et al. (69)]. It has also been shown to be upregulated in lung tissue-resident CD8 T-cells post influenza infection [Anthony et al. (70)].

In the GSVA plot for the CD8 naive subset (**Supplementary Figure 11A**), the Narcolepsy samples are upregulated in a gene set examining adjuvanted versus non-adjuvanted flu vaccine [Nakaya et al. (71)]. Narcolepsy samples are down-regulated in a gene set comparing naive versus Th17 enriched CD4 T-cells (GSE32901). This may imply that these naive CD8 T-cells may be proned to undergo a Tc17 differentiation program; CD8+ IL-17+ T-cells have been described in the context of inflammatory (auto-immune) disorders [Srenathan et al. (72)]. An inflammatory profile seems to be present in the “naive” subset of CD8 T-cells as was reported in the naive subset of CD4 T-cells.

#### CD8 effector subset

As was seen in the CD4 effector subset, the CD8 effector subset (**Figure 5B**; **Supplementary Figure 8**) exhibits a large number of differentially expressed lncRNAs. In contrast to the CD4 effector subset however, at least half of the effector CD8 differentially expressed lncRNAs are up-regulated in NAR samples. Two mitochondrial genes are upregulated in NAR samples: COX7A2L (cytochrome C oxidase subunit 7A2 like, **Figure 6D**) and NDUFB5 (NADH ubiquinone oxidoreductase subunit B5, **Figure 6D**) both involved in electron transport. Both of these genes are involved in the pathways of several neurodegenerative disorders such as Huntington’s, Alzheimer’s and Parkinson’s diseases.

FOXP3 (which plays a role in maintaining immunological tolerance) is upregulated in the NAR effector subset (**Figure 6D**). FOXP3 is transiently induced in human CD8 T-cells upon TCR activation [Gavin et al. (73)]. CD8+FOXP3+ T-cells have been described in literature for both mice and humans with clinical relevance in the treatment of AIDS [Churlaud et al. (74)].

In the GSVA plot for the CD8 effector subset (**Supplementary Figure 11B**), the GSE32901 gene set comparing Th1 versus Th17 is again present, indicating an inflammatory profile as reported in the CD8 naive subset. Narcolepsy samples are down-regulated in a gene set comparing double-positive (CD4+CD8+) versus CD4intCD8+ thymocytes (GSE13493). Elevated double-positive lymphocytes have been reported in disease settings [Hess et al. (75)]. It has been shown that antigen stimulation can induce reactive CD8 T-cells to differentiate into double-positive T-cell subsets, augmenting functional characteristics [Schad et al. (76)]. For our analysis, we selected either CD4+ or CD8+ based on subset gating (**Supplementary Figure 1**) and would not have double-positive Tcells in our analyses. The only CD8-specific gene set present in this GSVA plot is GSE26669 which compares untreated CD8 T-cells versus CD8 T-cells treated with leukocyte costimulatory blockade antibodies.

#### CD8 effector memory subset

FOXO3 is downregulated in the NAR effector memory (**Figure 5C**; **Figure 6E**) subset. It has been reported (in the CD4 compartment) that FOXO3 proteins control the differentiation of FOXP3 regulatory T-cells [Ouyang et al. (77)]. Mice with a conditional deletion of Foxo3 in T-cells developed reduced autoimmune encephalomyelitis [Stienne et al. (78)]. In CD8 T-cells, FOXO3 deficiency has been shown to increase the number of memory CD8 T-cells without affecting functional quality [Sullivan et al. (79)].

IL-6 (Interleukin-6, upregulated in NAR effector memory subsets (**Figure 6E**) is a cytokine that function in immunity and metabolism. The protein is produced at sites of acute or chronic inflammation, elevated levels of which have been found in viral infections (including, most recently, in COVID-19). Higher plasma levels of IL-6 in narcoleptic patients have been reported in previous studies [Tanaka et al. (80)] and IL-6 production has been shown (by mass cytometry) to be elevated in B-cells, but not CD8 T-cells [Hartmann et al. (14)]. IL-6 has been suggested to be a “sleep factor” [Vgontzas et al. (81)] with secretions that correlate with sleep and sleepiness. C-X-C motif chemokine receptor 1 (CXCR1, **Figure 6E**), also known as the IL-8 receptor, levels are markedly down-regulated (log_2_ -8) in samples from NT1 patients. Its ligand, IL-8, like IL-6, has been reported as elevated in the plasma of narcoleptic patients [Tanaka et al. (80)].

TTLL7 (tubulin-tyrosine ligase-like 7), upregulated in NAR CD8 effector memory subset (**Figure 6E**), is a polyglutamylase which preferentially modifies beta-tubulin and is involved in neurite growth and postnatal neuronal maturation. Polyglutamylation is a regulator of microtubule function; hyper-glutamylation may cause neuro-degeneration [Bodakuntla et al. (82)]. Post-translational modifications, such as glutamylation, are necessary for TCR activation [Martín-Cófreces et al. (83)].

In the GSVA plot for the CD8 effector memory subset (**Supplementary Figure 11C**), like the CD8 naive and effector GSVA plots, GSE32901 is present, indicating an inflammatory profile. Narcolepsy samples are down-regulated in the gene set comparing effector versus memory CD4 T-cells (GSE43863).

#### CD8 central memory subset

PTCH2 (Patched 2, upregulated in NAR, **Figure 5D**; **Figure 6F**) and CCND1 (Cyclin D1, down-regulated in NAR, **Figure 6F**) are both involved in the hedgehog signaling pathway [Katoh and Katoh (84)], which is a key developmental regulator. Mammals have three hedgehog homologues: desert, indian and sonic. PTCH2 gene product is one of the receptor for sonic hedgehog. CCND1 has been shown to be involved in immuno-suppression [Chen et al. (85)]. Hedgehog signaling requires a negative feedback loop involving PTCH2 and induces cellular proliferation involving CCND1 [Katoh and Katoh (84)]. The hedgehog signaling pathway plays a role in the differentiation, survival and proliferation of T-cells. It has been shown that the constitutive activation of the hedgehog pathway may lead to decreased TCR signal strength resulting in an increase in self-reactive T-cells as well as altered T-cell lineage decisions [Rowbotham et al. (86)].

The GSE320901 gene set (indicating an inflammatory profile) is not present in the GSVA plot for the CD8 central memory subset (**Supplementary Figure 11D**), as it was in the other CD8 T-cell subsets. In this subset, there is the presence of GSE33374, a gene set comparing CD161++CD8+ alpha/alpha T-cells versus CD161++CD8+ alpha/beta T-cells. CD161 is a marker of all IL17 producing T-cell subsets and has been implicated in auto-immune diseases [Konduri et al. (87)].

### Inflammatory genes

**Figure 7:**
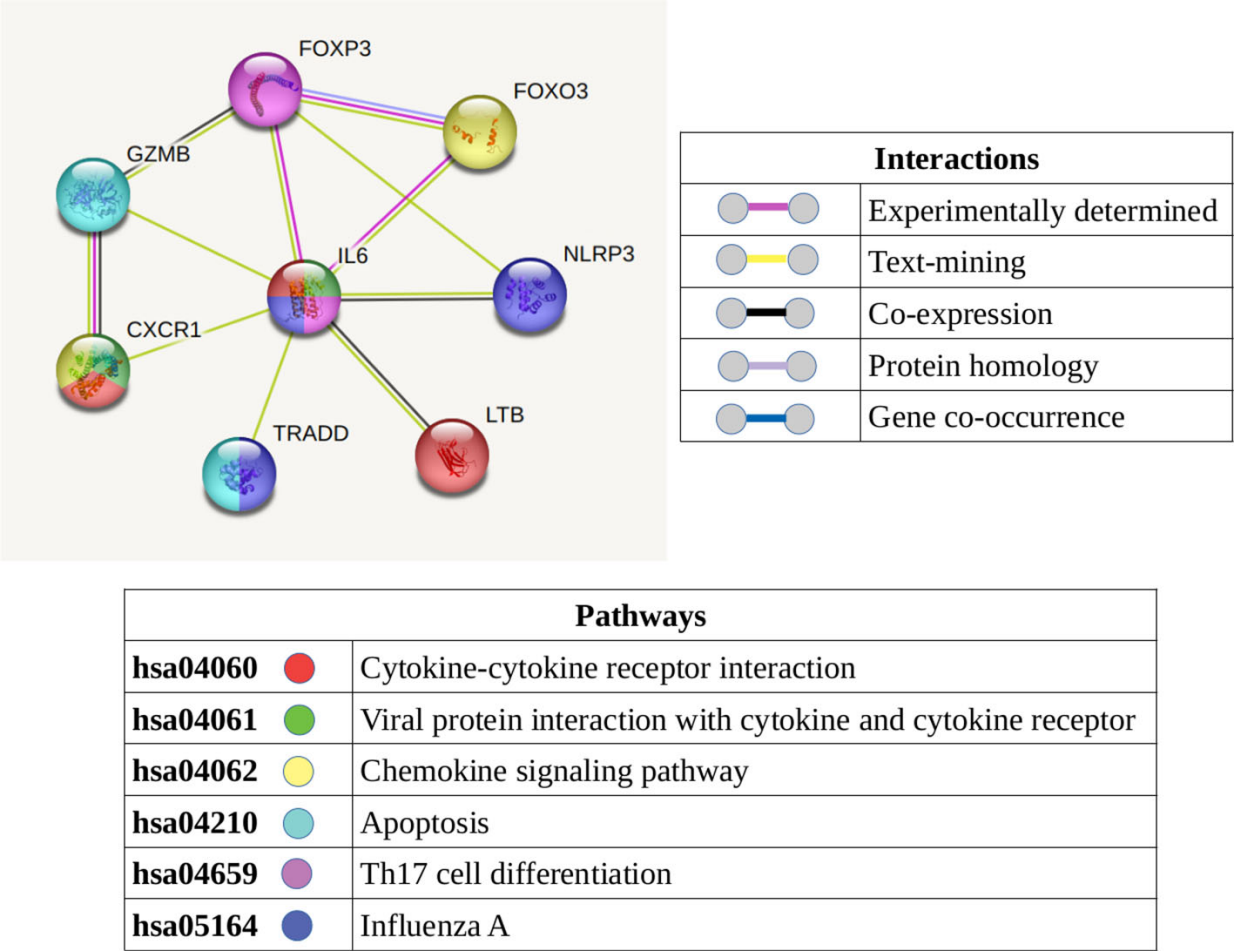
Protein-protein interaction network displaying the known and predicted interactions between the molecules (which are present and detected in the T-cells of narcolepsy patients) identified as involved in an inflammatory response. The color of the node denotes its involvement in the KEGG pathways listed while the color of the connecting lines summarize the interactions.

Inflammatory response genes were identified in both CD4 and CD8 T-cell subsets. Based on the protein-protein interactions of these inflammatory response genes, we can hypothesize multiple interactions between the CD4 (GZMB, LTB) and CD8 (IL6, CXCR1, TRADD, NLRP3, FOXO3, FOXP3) T-cell compartments (**Figure 7**). In addition to these inflammatory response genes, an inflammatory profile was identifies in both T-cell compartments via the GSVA analyses, most notably in the “naive” subsets.

## Conclusion

This transcriptomics analysis aimed to characterize blood CD4 and CD8 T-cell subsets and determine their potential contributions to narcolepsy development. We compared the transcriptome of NT1 patients against that of matched healthy controls and matched individuals suffering from other sleep disorders. We identified NT1-specific DEGs including several lncRNAs and pseudogenes in both the CD4 and CD8 compartments which will need to be characterized further.

We identified several genes involved in tubulin arrangement were identified in CD4 (*TBCB, CCT5, EML4, TPGS1, TPGS2)* and CD8 (*TTLL7*) T cell subsets, which play a role in the immune synapse formation and TCR signaling. Furthermore, we identified genes (*GZMB, LTB* in CD4 and *NLRP3, TRADD, IL6, CXCR1, FOXO3, FOXP3* in CD8) involved in various aspects of inflammation and the inflammatory response. We hypothesize that the tubulin arrangement genes may increase/decrease the specificity of the antigen recognition, which could lead to T-cell activation and enhance or prolong the inflammatory response.

The most striking observation was the identification of an inflammatory profile present in both CD4 and CD8 naive subsets. Although causal effect cannot formally be claimed, this observation suggests a possible involvement in the development or progression of the narcoleptic process. Further validation in samples from recent-onset narcolepsy patients may shed more light on these observations.

IFIT2 is upregulated in the CD4 effector subset while IFIT3 is upregulated in the CD4 central memory subset. The presence of these two type I interferon-induced genes may be a sign of an “effectorness gradient” (transcriptional progression from naive to central memory to effector memory) which has been shown to shape the response of CD4 T-cells to cytokines. Both IFIT2 and IFIT3 were reported to have increased expression upon iTreg (induced regulatory T-cells involved in immune tolerance) stimulation [Cano-Gamez et al. (88)].

Several of the genes (TBCB, CCT5, TTLL7, SGTB, NEFM, SYNGR3, NR4A2, ELP3, NQO2) identified are involved in neuronal projections. How the expression of these genes in T-cell subsets relate to the destruction of orexin-producing neurons in unknown. Their detection in the blood, however, may serve as potential biomarkers of disease development and/or progression.

Genes involved in the addiction pathway (FOS, FOSB, PPP1CA and RGS9) were identified in the CD4 subsets. Though it has been widely hypothesized that low orexin levels may affect the risk and reward seeking behavior of narcolepsy patients, little differences have been seen in studies comparing them to healthy controls [Dimitrova et al. (89); Barateau et al. (90)].

### Study limitations

The narcoleptic group included in this study was comprised of patients with a relative long delay from disease onset to diagnosis and sampling (an average of 72 months). An attempt to determine disease-specific immunological transcriptomic differences would require samples from patients with recent-onset (diagnosis within a few months), a real challenge with this rare and difficult to diagnose condition [Zhang et al. (91)]. A lapse of several years between disease onset and diagnosis (first blood draw) may dilute the effects of the immune system on the disease process due to the immunological history of each patient and may reveal changes that occur as a consequence, rather than a cause, of the disease process.

Human samples are, in general, highly variable in nature due to genetics, environment, lifestyle, immunological history, etc. A large sample size is a good way to filter out the noise that may result from these variables. We had a rather small (eleven subjects per condition) sample size which proved to be computationally demanding, given that we analyzed eight different highly purified T-cell subsets per individual. In this study, we only included narcolepsy patients with a definite diagnosis (after a complete sleep latency test, HLA genotyping, CSF measurement of orexin/hypocretin), thereby reducing intragroup heterogeneity. We minimized the effects of potential noise encountered by matching (age, sex and HLA) patients and controls as well as taking advantage of the robust features provided by the differential expression tool (edgeR) used [Zhou et al. (26)].

Goods et al. reported that blood handling (involving various storage and shipment conditions), prior to leukocyte isolation and sorting, impacts the global transcriptome of circulating immune cells [Goods et al. (92)]. Both NT1 and OSD blood samples were drawn and shipped overnight for processing while HD samples were drawn locally and processed within a few hours. In order to avoid any potentially confounding effects on the downstream transcriptomics analysis due to different blood handling and shipment conditions, both HD and OSD samples were used as controls for the NAR samples. This was also beneficial in identifying narcolepsy-specific DEGs as opposed to genes that may be differentially expressed due to the dysregulation of the sleep/wake cycle.

### Perspective

Validation of gene sets in recent-onset cases will determine whether the differential expression is the consequence of the narcoleptic process on T-cells or, rather, contributing to the disease progression. If the latter, genes or gene sets may be used as biomarkers to monitor disease progression.

Moreover, testing whether some genes or pathways, such as the perforin/granzyme B pathway, are directly involved in narcolepsy pathogenesis will require further studies. However, we have used our immune-mediated mouse model of narcolepsy [Bernard-Valnet et al. (93)] to assess the contribution of the T-cell cytotoxicity pathway in this pre-clinical model. It revealed that cytotoxic CD8 T-cells appear to be essential for the destruction of orexin-producing neurons.

The presence of a large proportion of differentially expressed lncRNAs may suggest a complex and context-dependent functionality, either contributing to or as a consequence of the disease. A more global analysis focusing on mRNA, lncRNA and miRNA using recently diagnosed cases to generate competing endogenous RNA (ceRNA) networks may shed some light on the intricacies of the disease and identify potential therapeutic targets.

## Data availability statement

The original contributions presented in the study are publicly available. This data can be found here: https://www.ncbi.nlm.nih.gov/geo/query/acc.cgi?acc=GSE240851.

## Ethics statement

The studies involving humans were approved by Comité de Protection des Personnes (2018-A00703-52, SOMNOBANK). The studies were conducted in accordance with the local legislation and institutional requirements. Written informed consent for participation in this study was provided by the participants’ legal guardians/next of kin.

## Author contributions

X-HN and RL developed the experimental design. X-HN, CQ and MC performed FACS sorting and quality controls. LB and YD provided the samples and clinical information. LK performed the computational analysis. LK wrote the manuscript. MZ and RL provided support, guidance and direction of the analysis and reviewed/edited the manuscript. All authors contributed to the article and approved the submitted version.

## Supplementary material

Supplementary Figure 1 T-cell gating and sorting summary with average cell counts **(A)**. Left: CCR7/CD45RA staining gated on CD4+ CD3+ live cells prior to cell sorting. Right: CCR7/CD45RA on each of the four CD4 T-cell subsets following the cell sorting. **(B)**. Average cell counts and isolated RNA concentrations for each CD4 T-cell subset sorted from PBMCs of narcolepsy, healthy donor and other sleep disorder individuals. **(C)**. Left: CCR7/CD45RA staining gated on CD8+ CD3+ live cells prior to cell sorting. Right: CCR7/CD45RA on each of the four CD8 T-cell subsets following the cell sorting. **(D)**. Average cell counts and isolated RNA concentrations for each CD8 T-cell subset sorted from PBMCs of narcolepsy, healthy donor and other sleep disorder individuals.
