# Supplementary figures and images for: The transcriptomics profiling of blood CD4 and CD8 T-cells in narcolepsy type I
Source: Front Immunol. 2023 Nov 23;14:1249405. doi: 10.3389/fimmu.2023.1249405 (PMC10702585; doi:10.3389/fimmu.2023.1249405)

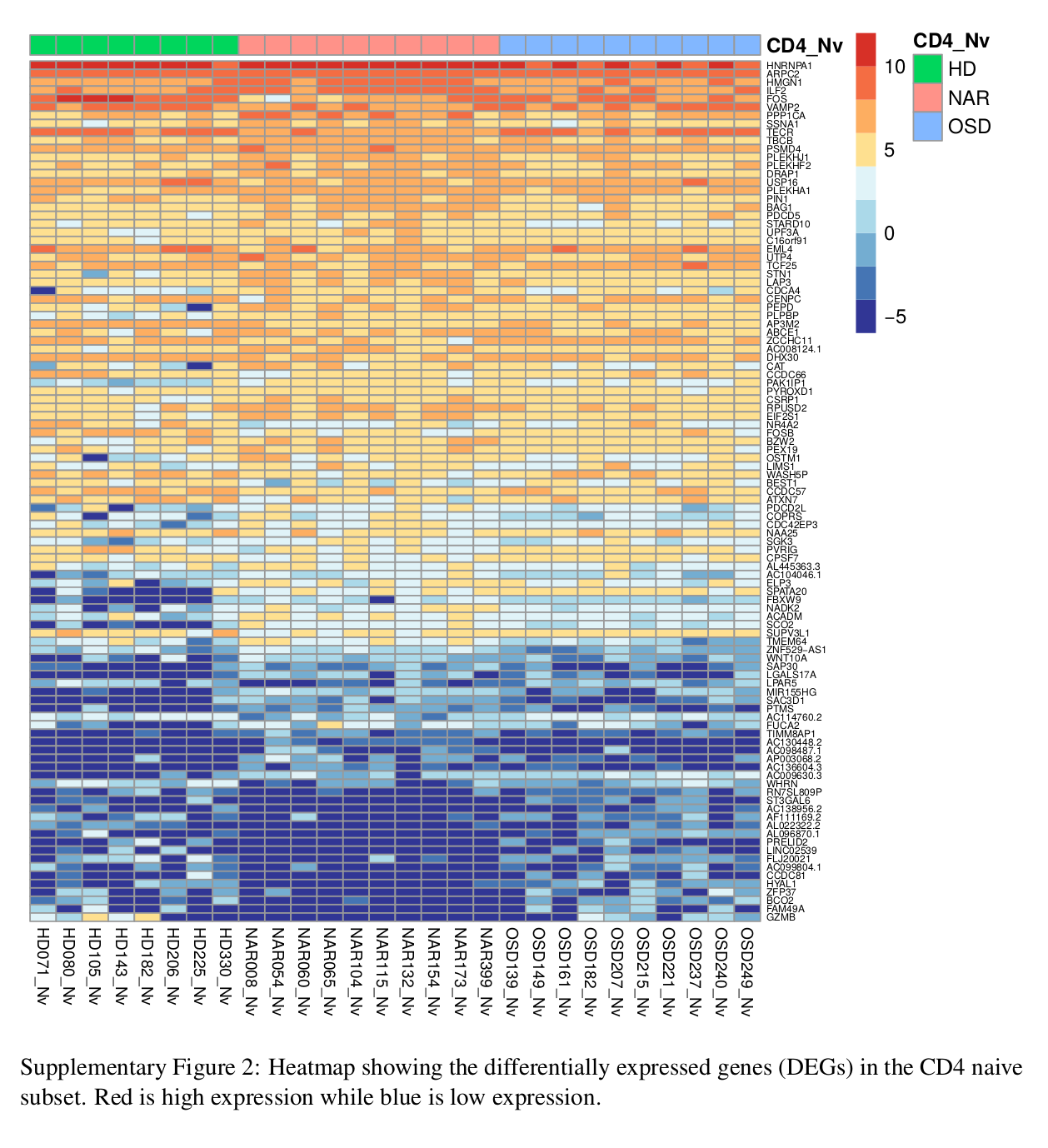

Supplement: Supplementary Figure 1 — T-cell gating and sorting summary with average cell counts (A). Left: CCR7/CD45RA staining gated on CD4+ CD3+ live cells prior to cell sorting. Right: CCR7/CD45RA on each of the four CD4 T-cell subsets following the cell sorting. (B). Average cell counts and isolated RNA concentrations for each CD4 T-cell subset sorted from PBMCs of narcolepsy, healthy donor and other sleep disorder individuals. (C). Left: CCR7/CD45RA staining gated on CD8+ CD3+ live cells prior to cell sorting. Right: CCR7/CD45RA on each of the four CD8 T-cell subsets following the cell sorting. (D). Average cell counts and isolated RNA concentrations for each CD8 T-cell subset sorted from PBMCs of narcolepsy, healthy donor and other sleep disorder individuals. [file Image_1.tiff]

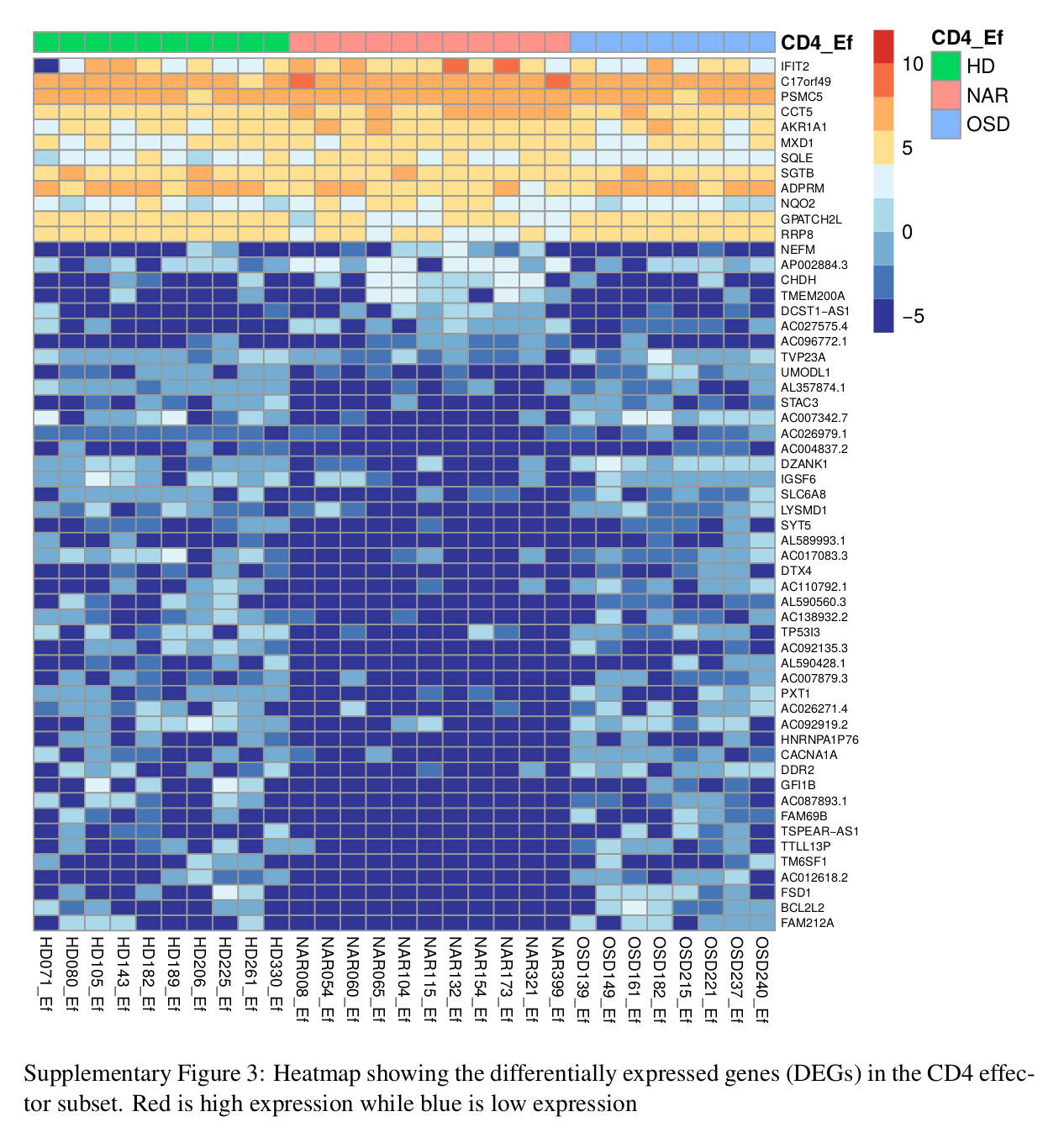

Supplement: Supplementary file 2 [file Image_2.tiff]

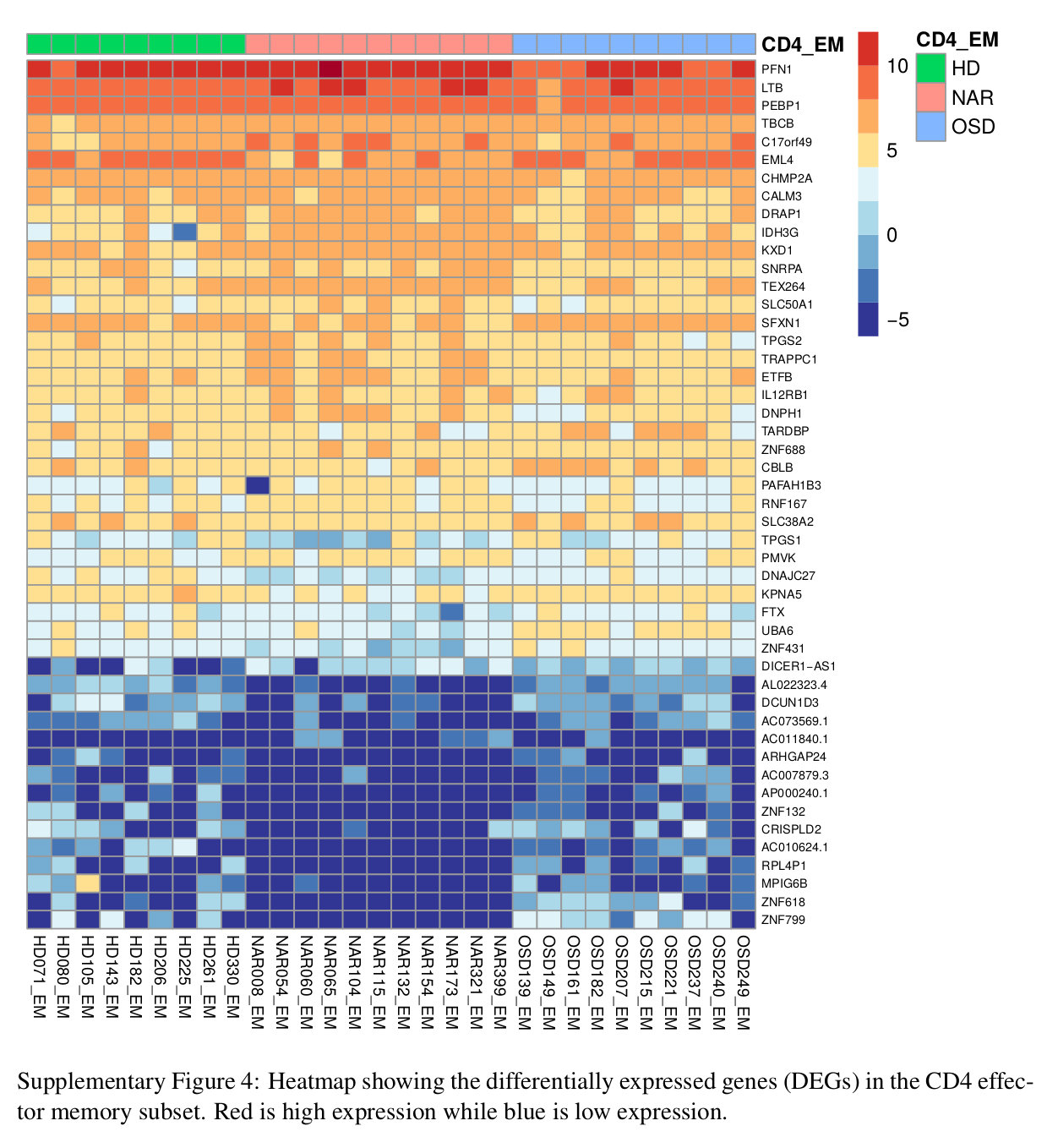

Supplement: Supplementary file 3 [file Image_3.tiff]

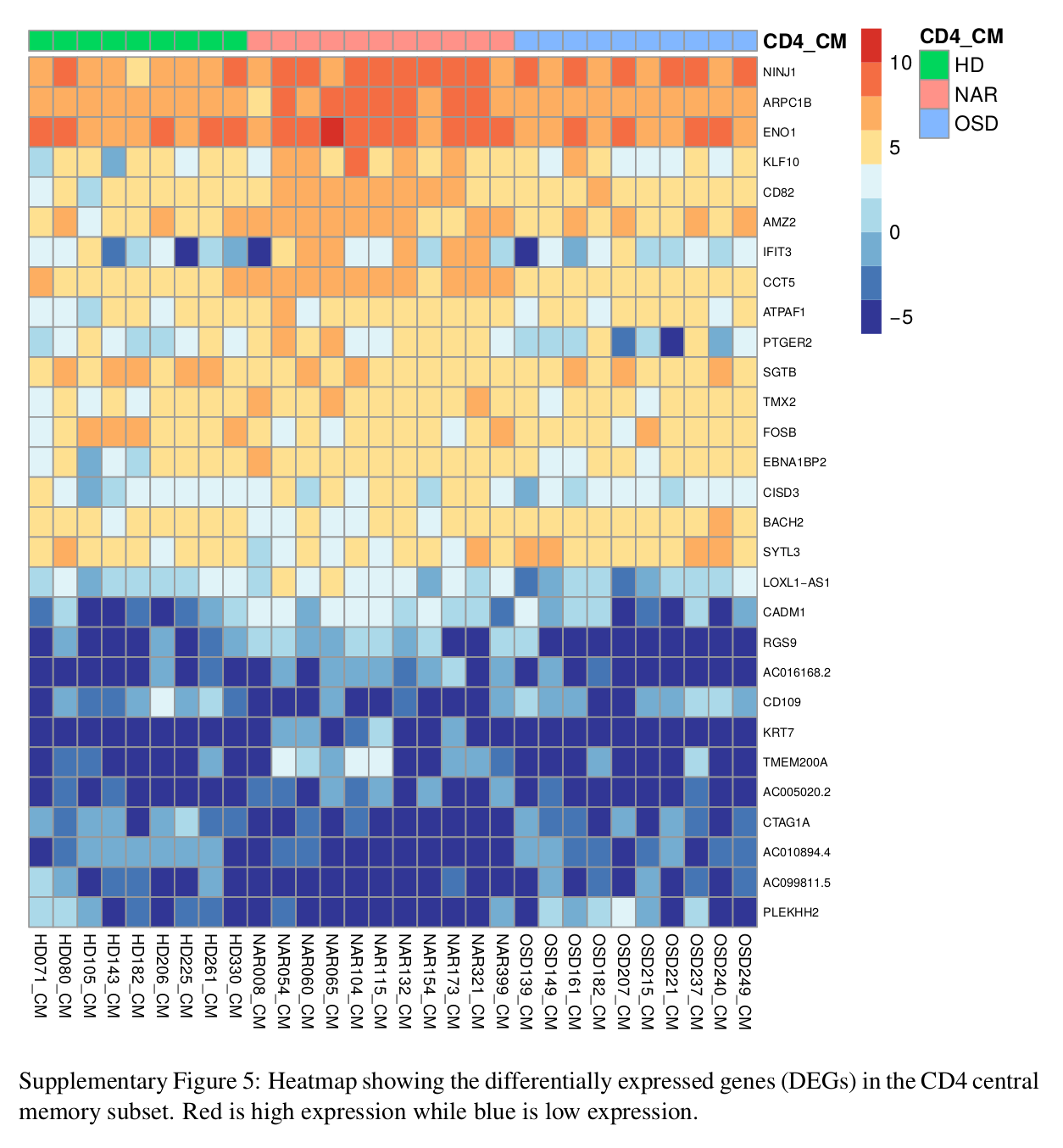

Supplement: Supplementary file 4 [file Image_4.tiff]

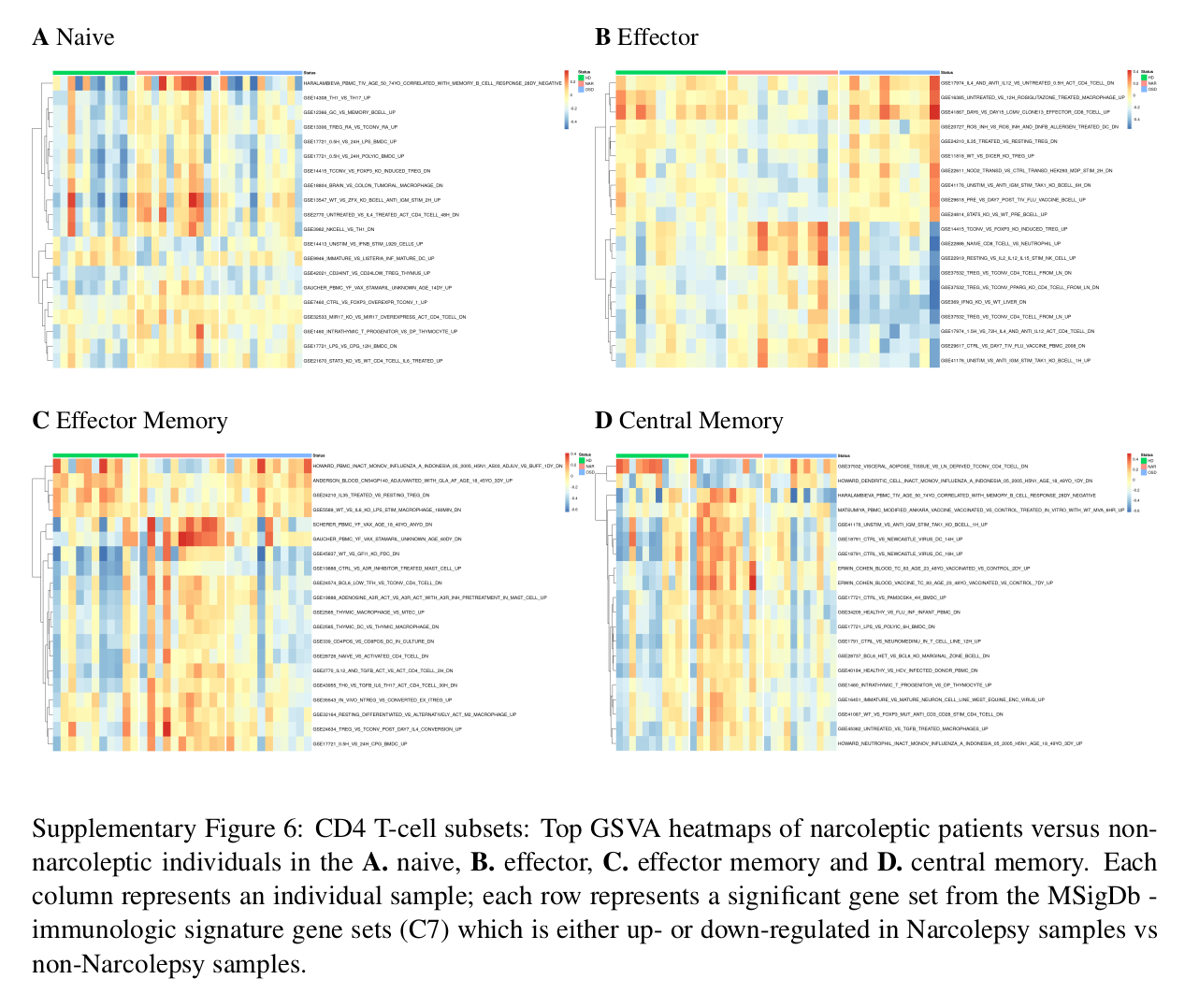

Supplement: Supplementary file 5 [file Image_5.tiff]

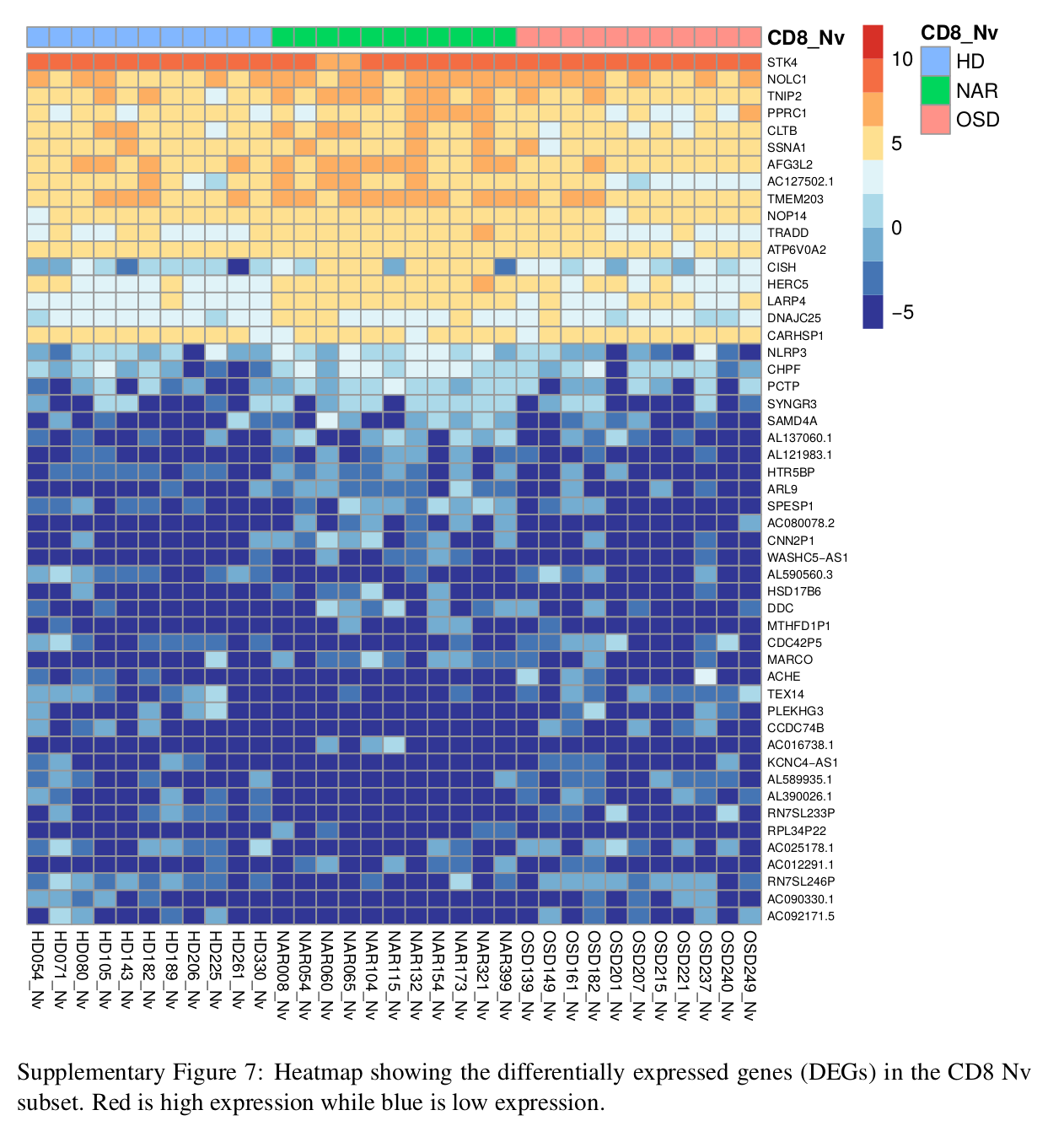

Supplement: Supplementary file 6 [file Image_6.tiff]

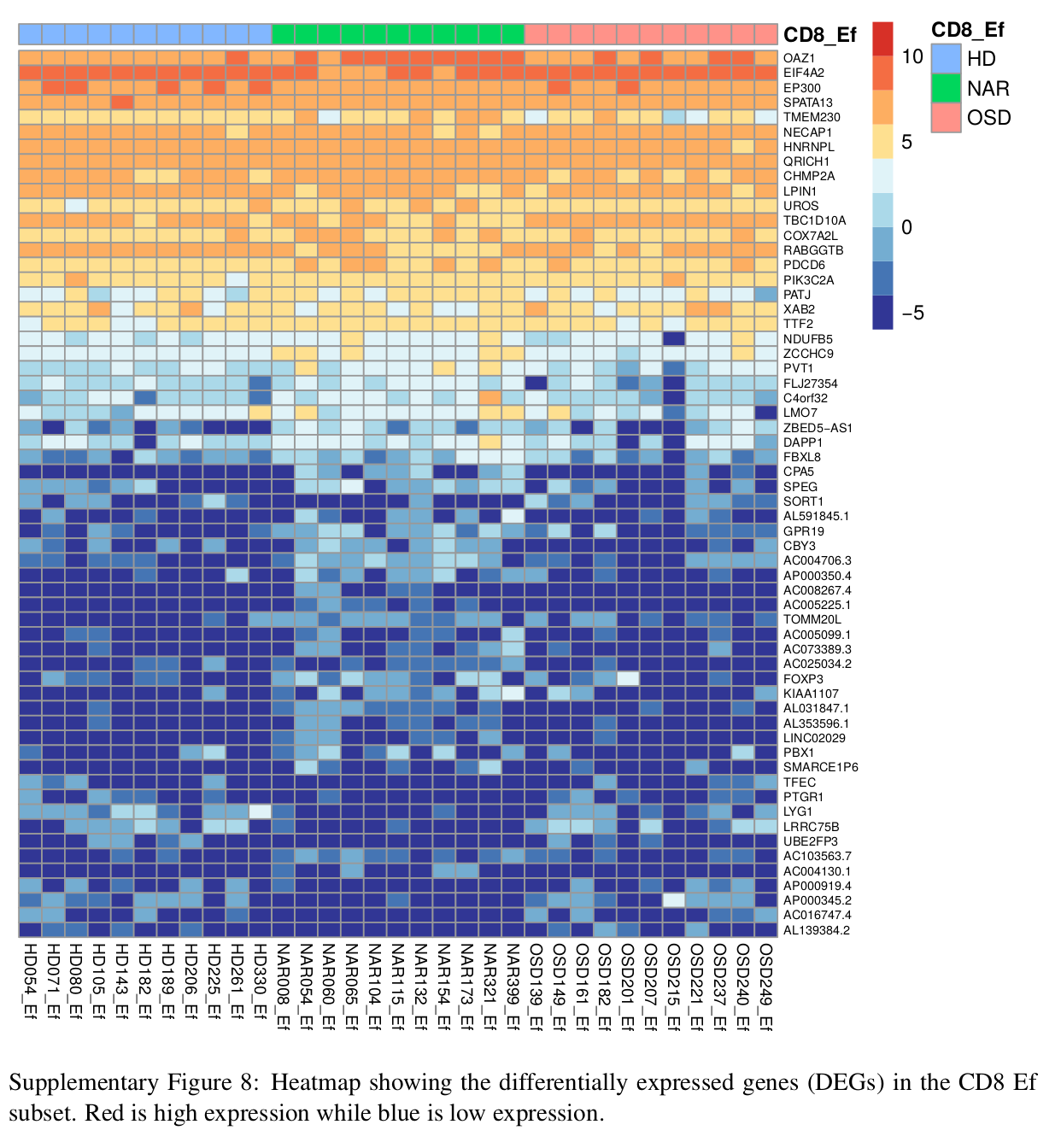

Supplement: Supplementary file 7 [file Image_7.tiff]

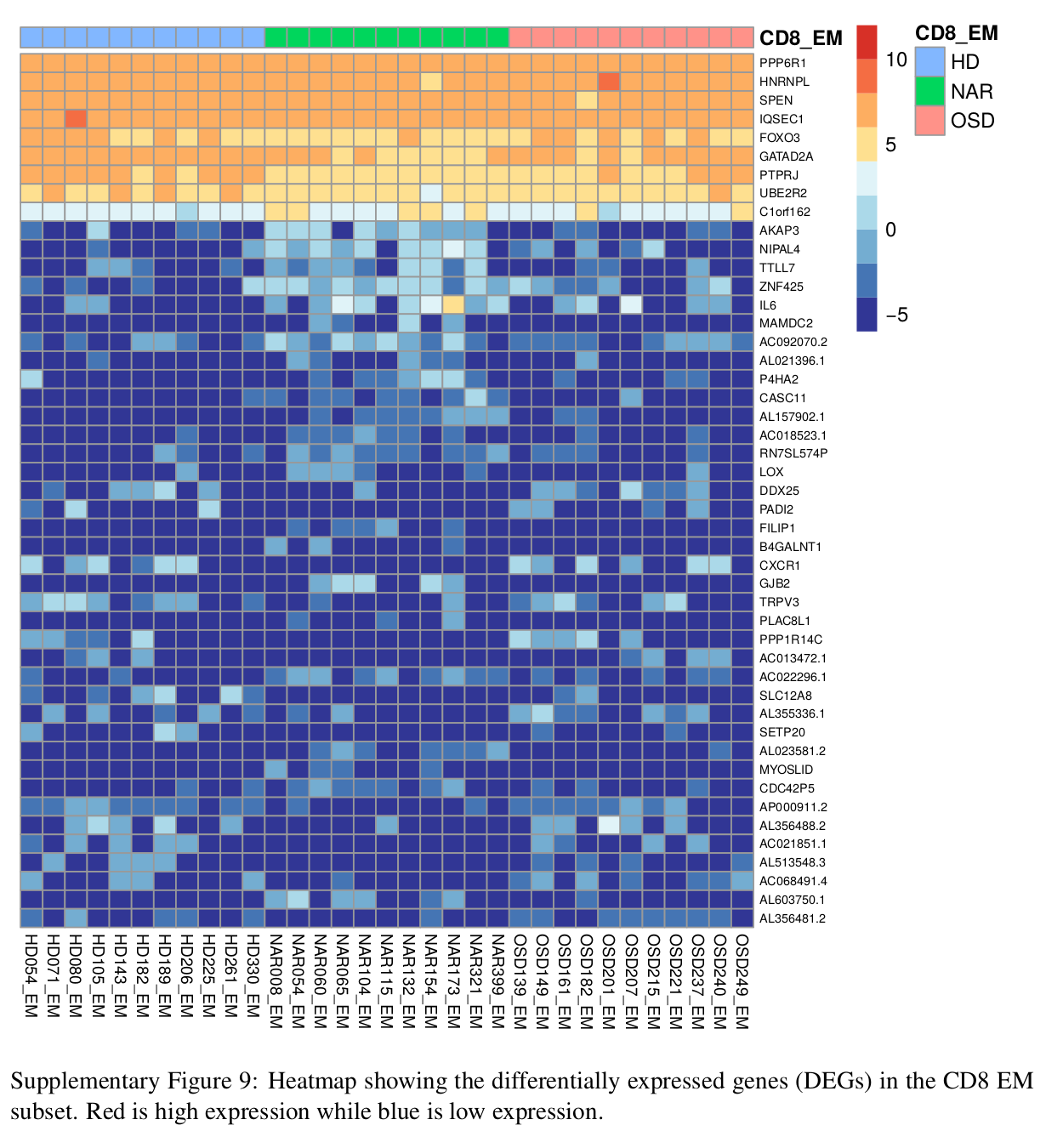

Supplement: Supplementary file 8 [file Image_8.tiff]

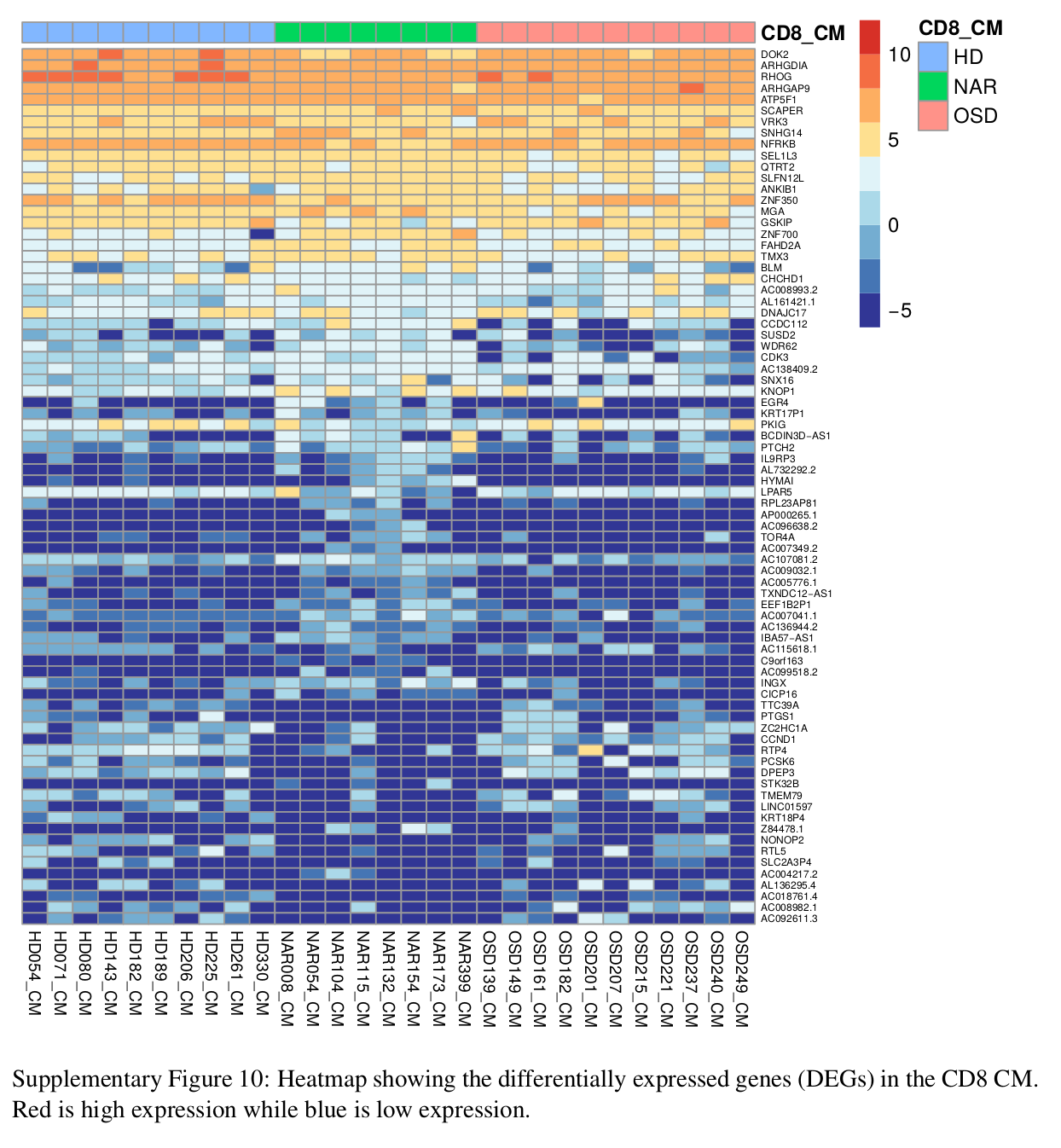

Supplement: Supplementary file 9 [file Image_9.tiff]

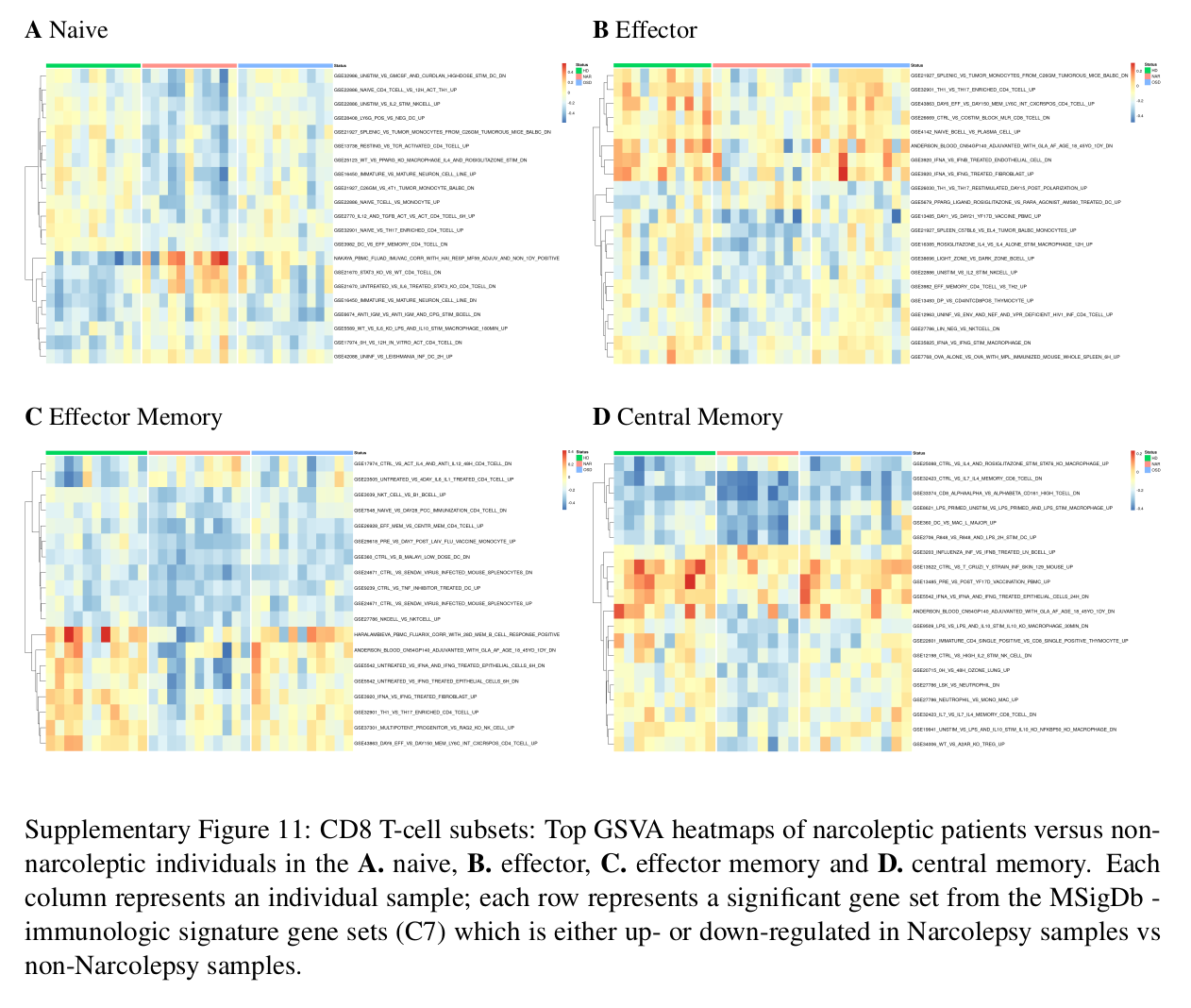

Supplement: Supplementary file 10 [file Image_10.tiff]
